# Transcriptome profiling of the rat retina after optic nerve transection

**DOI:** 10.1038/srep28736

**Published:** 2016-06-29

**Authors:** Masayuki Yasuda, Yuji Tanaka, Kazuko Omodaka, Koji M. Nishiguchi, Orie Nakamura, Satoru Tsuda, Toru Nakazawa

**Affiliations:** 1 Department of Ophthalmology, Tohoku University Graduate School of Medicine, 1-1 Seiryo-machi, Aoba-ku, Sendai, Miyagi, 980-8574, Japan; 2 RIKEN Center for Life Science Technologies (Division of Genomic Technologies), 1-7-22 Suehiro-cho, Tsurumi-ku, Yokohama, Kanagawa, 230-0045, Japan; 3 Department of Advanced Ophthalmic Medicine, Tohoku University Graduate School of Medicine, 1-1 Seiryo-machi, Aoba-ku, Sendai, Miyagi, 980-8574, Japan; 4 Department of Retinal Disease Control, Tohoku University Graduate School of Medicine, 1-1 Seiryo-machi, Aoba-ku, Sendai, Miyagi, 980-8574, Japan

## Abstract

Glaucoma is a group of eye diseases characterized by alterations in the contour of the optic nerve head (ONH), with corresponding visual field defects and progressive loss of retinal ganglion cells (RGCs). This progressive RGC death is considered to originate in axonal injury caused by compression of the axon bundles in the ONH. However, the molecular pathomechanisms of axonal injury-induced RGC death are not yet well understood. Here, we used RNA sequencing (RNA-seq) to examine transcriptome changes in rat retinas 2 days after optic nerve transection (ONT), and then used computational techniques to predict the resulting alterations in the transcriptional regulatory network. RNA-seq revealed 267 differentially expressed genes after ONT, 218 of which were annotated and 49 unannotated. We also identified differentially expressed transcripts, including potentially novel isoforms. An *in silico* pathway analysis predicted that CREB1 was the most significant upstream regulator. Thus, this study identified genes and pathways that may be involved in the pathomechanisms of axonal injury. We believe that our data should serve as a valuable resource to understand the molecular processes that define axonal injury-driven RGC death and to discover novel therapeutic targets for glaucoma.

Glaucoma is the second most common cause of blindness worldwide^1^. The key contributor to glaucoma progression is elevated intraocular pressure (IOP)^2^, and the current standard treatment for glaucoma is reducing IOP^3^. However, even substantial IOP reduction cannot prevent the development and progression of glaucoma in many clinical cases. The limits of glaucoma treatment thus call for the investigation of IOP-independent pathomechanisms of retinal ganglion cell (RGC) death.

The optic nerve head in patients with glaucoma is characterized by distinctive morphological changes, of which the most important is cupping. Visual field defects in glaucoma patients correspond to the area of this cupping^2^. The axon bundles in the cupped area have been observed to be mechanically stressed, which is likely associated with axonal injury^4^ and eventual RGC death^5^. Therefore, axonal injury has been proposed as an IOP-independent factor contributing to the pathogenesis of glaucoma. However, little is known about the molecular events that link axonal injury to RGC death. Ultimately, therefore, in order to prevent RGC death and reduce blindness due to glaucoma, it will be necessary to uncover the molecular changes that follow axonal injury and identify therapeutic targets for neuroprotective drugs.

One of the most useful approaches to understanding global molecular events in the retina under pathological conditions is the comprehensive analysis of the transcriptome in animal models of disease. Techniques for transcriptome analysis are mainly based on hybridization or sequencing approaches^6^. The microarray method, which uses the hybridization approach, is a well-established technique, and is widely used for investigating pathomechanisms in various diseases, including neurodegenerative diseases^7^. In the field of ophthalmology, microarray-based transcriptome analysis of animal models of axonal injury has revealed many candidate genes for the pathomechanism of RGC death^7^,^8^. However, the microarray method has several limitations. It relies on probes that have been designed based on known sequences, it can be affected by background fluorescence due to cross-hybridization (which impairs the detection of lowly expressed genes), and it has a limited dynamic range due to saturation of the hybridization signal^6^.

RNA sequencing (RNA-seq) is a relatively new technique that is gradually gaining popularity. It uses a sequencing-based approach, does not require pre-designed probes and can detect and quantify entire transcripts, including unknown ones^6^. Moreover, RNA-seq has the advantage of being able to detect splice isoforms^9^. Thus, RNA-seq promises to improve our understanding of global gene expression changes. Previously, we used RNA-seq^10^ and another new technique, cap analysis of gene expression (CAGE)^11^, to analyze transcriptome changes in the mouse retina after optic nerve crush (ONC). These techniques were effective in identifying numerous genes that may be involved in the pathomechanism of axonal injury after ONC, but the large number of candidate genes made it difficult to characterize their roles in detail and to select the most promising therapeutic targets. Thus, in the present study, we set out to identify the most promising of these previously identified differentially expressed genes (DEGs) by analyzing changes in a similar, but different axonal injury model and comparing the results. For the new injury model, we chose optic nerve transection (ONT) in rats, because these animals are commonly used to study axonal injury, and because post-ONT retinal changes have both similarities and differences with post-ONC changes^8^. RGC death is faster after ONT^12^,^13^, and, crucially, DEGs after ONT differ from those after ONC^8^. While the fundamental biological processes leading to RGC death are likely similar in these two axonal injury models^14^, we expected that gene expression changes not directly related to RGC death would differ between them.

Thus, in this study, we used RNA-seq to analyze retinal samples taken 2 days after ONT in rats, in order to identify changes in comprehensive gene expression in the retina after axonal injury. We also used a pathway analysis to determine which transcriptional regulatory networks mediated RGC death after axonal injury.

## Results

### RNA-seq and differential gene expression analysis

To investigate the pathomechanisms of RGC death induced by axonal injury, we performed RNA-seq analysis of retinal samples extracted 2 days after ONT or a sham operation. Previously, it has been reported that RGC death (i.e., significant loss of total RGC numbers) starts after more than 3 days following ONT in rats, and that the structure of the RGCs is preserved before this point^15^,^16^,^17^. On the other hand, it has also been reported that glial activity, which is involved in neuronal inflammation, increases in the early stages after axonal injury, contributing to RGC death^18^. Furthermore, apoptotic events such as caspase-3 activation have been reported to occur at this stage^14^. Thus, examining the retinas at this time point allowed us to observe early molecular events preceding RGC death after ONT. For both groups, triplicate RNA samples were prepared from independent retinal RNA samples, each of which was derived from the retinas of 3 different rats. Before preparing the triplicate RNA samples, we performed quantitative real-time PCR (qRT-PCR) on each component sample, selecting 5 typical RGC markers (including the deprecated *rCG37080*: Rn01753495_m1 marker) to confirm that RGC injury had been successful in the ONT group. We used the delta delta Ct (ddCt) method to evaluate downregulation of these 5 RGC markers in the ONT samples^19^. First, for each RGC marker, we calculated dCt, i.e., we normalized the expression level of the RGC marker to a known-stable housekeeping gene (*Gapdh*). We used the following formula: RGC marker Ct – *Gapdh* Ct. Next, we calculated ddCt for each RGC marker in each sample, i.e., we normalized the expression level of the RGC marker to its expression in the sham group. We used the following formula: dCt – mean dCT of the sham group. We found that the mean summed ddCt of the RGC markers (mean ddCtRGC in Table S1) was higher in the ONT group than the sham group, indicating that their expression was reduced (Table S1). In addition, the summed ddCt of 5 RGC markers (ddCtRGC) in each ONT sample was higher than the mean + 3SD of the summed ddCt of the 5 RGC markers (mean + 3SD of ddCtRGC) in the sham group (Table S1), indicating that axonal injury-driven RGC damage was successfully induced, and that it was exclusively induced in the ONT group. After the initial validation, we also revalidated *Pouf4f1* mRNA expression levels using the updated Taqman probe for *Pou4f1* (ID: Rn01465571_m1). This revalidation confirmed that *Pouf4f1* mRNA expression in the ONT group was significantly downregulated (Fig. S1).

The quality of each sample used for RNA-seq was assessed with the Bioanalyzer device. RNA integrity measured with the Bioanalyzer ranged from 8.7 to 9.2 (Table S2), and thus the quality was judged high enough to yield reliable RNA-seq results. Sequencing was performed using the Illumina Hiseq2500. The sequence statistics are summarized in Table S2. The total number of reads per lane was about 382 million, and the total number of reads per sample ranged from 56.1 million to 77.3 million. The mapping rate of the RNA-seq reads ranged from 80.8 to 83.0%.

**Figure 1:**
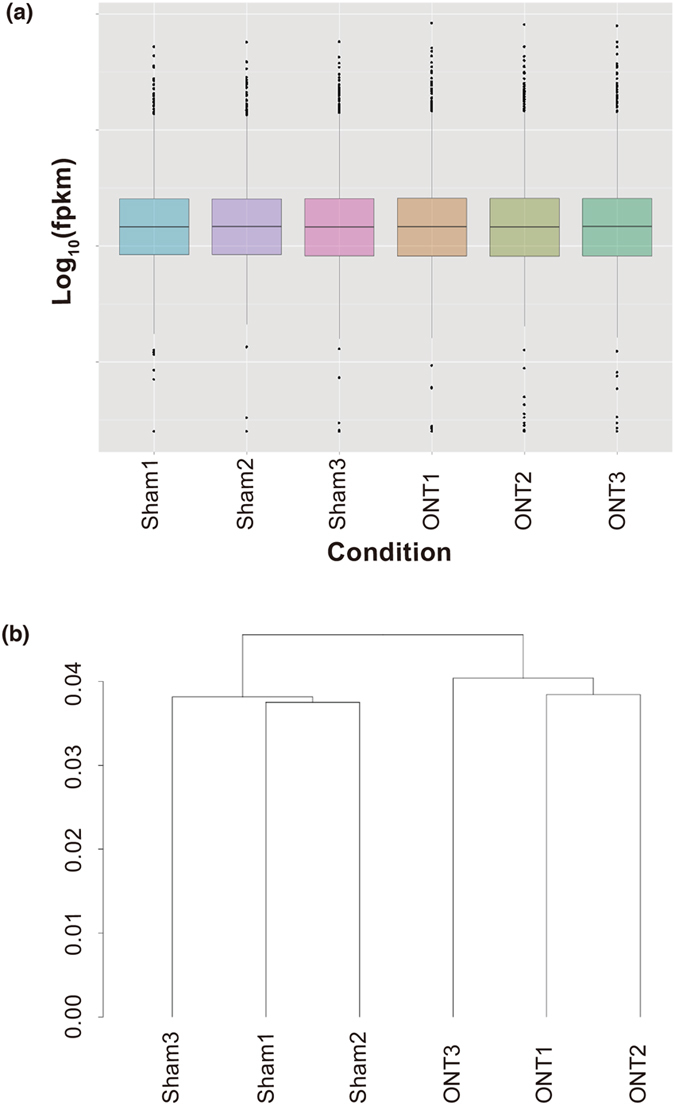
Boxplot and hierarchy of clusters showing gene expression level and pattern in each sample. The boxplot (**a**) shows the overall range and distribution of the gene expression level (log_10_ FPKM) in each sample. The dendrogram (**b**) shows the results of a hierarchical clustering analysis of gene expression levels (FPKM) in each sample.

In order to evaluate the dynamic range of the fragments per kilobase of exon per million fragments mapped (FPKM)^20^ values, representing the relative abundance of gene expression, we created boxplots of log_10_-transformed FPKM values for each replicate with the CummeRbund software^21^ (Fig. 1a). The overall range and distribution of the FPKM values were consistent among the samples, indicating that our RNA-seq data were reproducible and of high quality. We also created a dendrogram showing hierarchical clustering based on the transcript expression levels with CummeRbund (Fig. 1b). The dendrogram showed that the ONT samples were clearly separated from the sham samples, confirming that our RNA-seq data met the conditions for a differential expression analysis.

To extract candidate genes associated with pathomechanisms of axonal injury, we performed a differential expression analysis that compared the ONT and sham groups 2 days after surgery, using the Cuffdiff software^22^. We used only genes with mean FPKM > 0.1 in each group for this analysis to avoid inclusion of genes with low expression that are difficult to distinguish from sequencing noise^23^. Consequently, we identified 267 DEGs, which included 218 annotated and 49 unannotated genes (Table S3). The 20 most significantly upregulated annotated DEGs after ONT are shown in Table 1. The top upregulated genes among these 20 DEGs included endothelin-converting enzyme-like 1 (*Ecel1)*, Caspase 4, an apoptosis-related cysteine peptidase (*Casp4*), and Interferon alpha-inducible protein 27-like 2B (*Ifi27l2b*).

In order to narrow down the most promising candidate DEGs, we compared the 218 annotated post-ONT DEGs identified in this study with the results of a previous study, in which we identified 177 post-ONC DEGs in mouse retinas^10^. We found that 35 DEGs were upregulated in both models (Table S4), including 8 DEGs that were among the top 20 upregulated DEGs after rat ONT: *Ecel1*, *Tnfrsf12a*, *Gpnmb*, *Lgals3*, *Hmox1*, *Csrnp1*, *Sox11*, and *Chac1* (these overlapping DEGs are indicated with an asterisk [*] in Table 1). With the exception of *Hmox1*^24^, the role of these genes in axonal injury-induced damage has not yet been described in detail.

The 20 most significantly downregulated annotated DEGs after ONT are shown in Table S5. Ten of these 20 DEGs overlapped with the DEGs of the mouse ONC model. Referring to the microarray database of adult mouse retinal cell types^25^, we found that six of the ten DEGs (*Rasgrp2, Htr1b, Tppp3, Rbpms2, Pou4f2, and Kcnd2*) were expressed specifically in RGCs.

In the current RNA-seq data, we also identified 294 differentially expressed transcripts, including 103 potentially novel isoforms, with the Cufflinks program^22^. (Table S6). Five of the 20 most upregulated genes (indicated by the ^†^ mark in Table 1) also included differentially expressed transcripts that were potentially novel isoforms.

### Validation of DEGs with qRT-PCR

**Figure 2:**
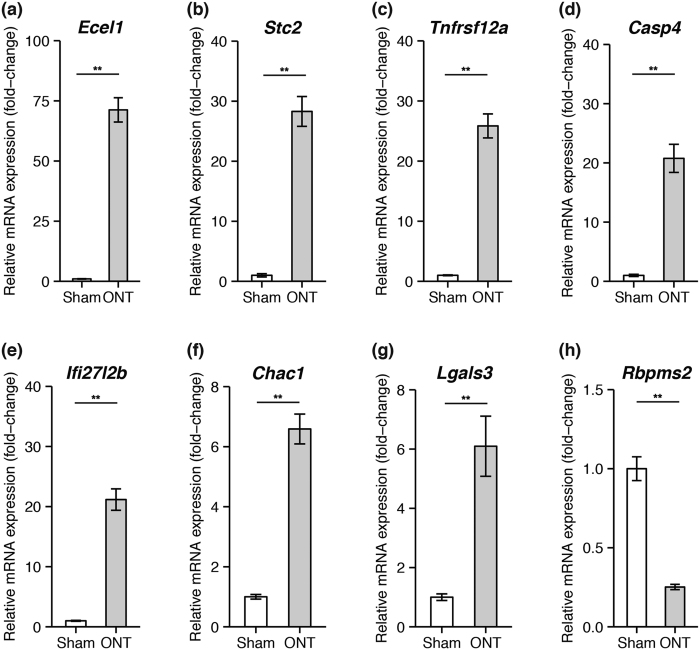
Validation of selected DEGs with qRT-PCR. Transcript expression changes in eight selected DEGs were validated with qRT-PCR. The graph shows mRNA expression in the ONT and sham groups, normalized to an average expression of 1.0 in the sham group. Values are mean ± S.E.M. (n = 9 in each group, **P < 0.01).

To evaluate the reliability of the RNA-seq findings, we selected eight DEGs (*Ecel1*, *Stc2, Tnfrsf12a*, *Casp4*, *Ifi27l2b*, *Chac1*, *Lgals3*, and *Rbpms2*) and examined them with qRT-PCR. *Rbpms2* is a specific RGC marker^26^, and was downregulated in both the rat ONT and mouse ONC models (Table S5). The other seven DEGs were highly upregulated after ONT. An analysis with conventional qRT-PCR showed a significant difference in the expression of these eight genes in the sham-treated and ONT samples (Fig. 2). This was consistent with the differential expression patterns observed in the RNA-seq data.

### *In silico* functional analysis of the DEGs

In order to investigate which biological functions were important after axonal injury, we performed an *in silico* functional analysis of the 218 DEGs with Ingenuity Pathway Analysis (IPA) software^11^. This showed that “cell-to-cell signaling and interaction” was the most significant biological function after ONT (Table 2). In this study, “cell-to-cell signaling and interaction” involved 73 DEGs, including *Tnfrsf12a*, *Mmp19*, *Flnc*, *Lgals3*, *Arc*, *Hmox1*, and *Lcn2*. These genes were also among the top 20 upregulated DEGs (Table 2). The second most significant function was “cell death and cell survival”, which involving 80 DEGs, including the following top-20-ranked genes: *Ecel1*, *Casp4, Tnfrsf12a*, *Lgals3*, *Hspb1*, *Arc*, *Hmox1*, *Csrnp1*, *Lcn2 and Sox11*. The following five genes were involved in both the most and second-most significant functions: *Tnfrsf12a*, *Lgals3*, *Arc*, *Hmox1* and *Lcn2*.

### Upstream regulator analysis

**Figure 3:**
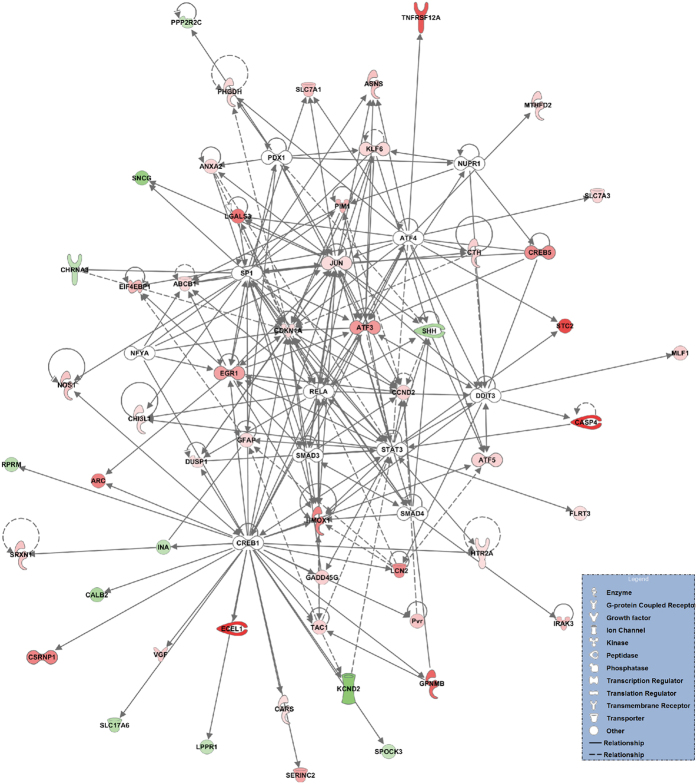
Predicted transcriptional regulatory networks affected by ONT IPA was used to predict upstream regulators. The predicted upstream transcription regulators (white nodes) were CREB1, ATF4, STAT3, RELA, DDIT3, SMAD4, SP1, PDX1, SMAD3, EGR1, NUPR1, and NFYA. The target molecules of these upstream transcription regulators are also shown, in order to illustrate alterations in the interaction network after ONT. The solid lines represent a direct interaction between two genes, whereas the dotted lines represent an indirect relationship. The length of the lines reflects the strength of reported evidence supporting the node-to-node relationship. The shapes of the nodes represent the different known biological roles of each of these molecules, as shown in the lower right inset. Red indicates upregulated genes and green indicates downregulated genes.

In order to identify the key upstream regulators governing molecular changes after ONT, we performed an upstream regulator analysis with IPA^27^. This identified 12 significant upstream transcription regulators that may play a role in mediating downstream molecular events after ONT (Table 3). Figure 3 shows a graphical representation (generated by merging the 12 upstream transcription regulators and the target molecules from the dataset) of the molecular networks that may have important roles after ONT. The most significant upstream transcription regulator was cAMP responsive element binding protein 1 (CREB1). The downstream target genes of CREB1 included *Ecel1*. Additionally, ATF4 and DDIT3, which are known to be activated during ER stress^28^, were predicted to be upstream regulators, and nuclear protein 1 (NUPR1), which is involved in various biological functions^29^, was predicted to activate several DEGs after ONT.

## Discussion

In this study, we conducted an RNA-seq analysis of rat retinas 2 days after the animals had undergone ONT, in order to profile the retinal transcriptome following axonal injury. This revealed a number of novel DEGs that may be involved in axonal injury, as well as differentially expressed transcripts that may include potentially novel isoforms. Furthermore, a subsequent *in silico* pathway analysis revealed significant upstream regulators and the comprehensive transcriptional regulatory networks orchestrated by these regulators.

Changes in the transcriptome are thought to be associated with RGC death, a key pathology in glaucoma, and our goal was thus to identify characteristic DEGs after axonal injury. In the field of ocular research, Oyuna S *et al*. have previously used RNA-seq to analyze rat retinal samples^30^. Their study used OXYS rats, which have a spontaneously-developed phenotype that resembles a number of human geriatric disorders. These rats had several DEGs and biological pathways related to AMD-like retinopathy. Other research aimed at elucidating the pathogenesis of axonal injury and discovering new neuroprotective drugs has often used animal models based on ONT in rats^31^,^32^,^33^. Our report builds on this previous research, in addition to research based on microarray analysis^8^,^34^, by using a rat ONT model and RNA-seq to analyze the retinal transcriptome after axonal injury.

We identified 267 DEGs, including 218 annotated genes. Among these DEGs, 35 were upregulated in both the rat ONT and mouse ONC models, of which 8 were identified as being among the top 20 DEGs after rat ONT. These genes were *Ecel1*, *Tnfrsf12a*, *Gpnmb*, *Lgals3*, *Hmox1*, *Csrnp1*, *Sox11*, and *Chac1*. The upregulation of *Ecel1* and *Gpnmb* was particularly interesting. *Ecel1*, which was the top upregulated gene after ONT, has been reported to promote antioxidant activity and act as a neuroprotective agent in neurons^35^. This suggests that *Ecel1* may play a role in the endogenous protective effect against axonal injury in the early stages after ONT. *Gpnmb* was initially identified and cloned from highly metastatic melanoma cells and was found to promote tumor growth^36^. It has also been reported that mutations in GPNMB protein in DBA/2J mice contribute to pigmentary glaucoma^37^. Nevertheless, GPNMB has also been reported to have a beneficial role, promoting neuroprotection in animal models of amyotrophic lateral sclerosis^38^ and cerebral ischemia-reperfusion injury^39^. In these models, GPNMB was found to act via phosphorylation of ERK1/2 and Akt. Thus, the *Gpnmb* gene is a promising new therapeutic target for the prevention of axonal injury-driven RGC death.

Another significant finding was that ChaC, cation transport regulator 1 (*Chac1*) was highly upregulated after ONT. We previously reported that *Chac1* mRNA was upregulated in mouse retinas after ONC^10^. CHAC1 protein degrades glutathione and sensitizes cells to oxidative stress^40^, which we found played a critical role in axonal injury-induced RGC death^41^. We thus suggest that *Chac1* is involved in axonal injury-induced RGC death and is a promising, novel therapeutic target for neuroprotection in glaucoma treatment.

Interestingly, we found some genes that responded differently in the rat ONT and mouse ONC models, including *Casp4* and *Ifi27l2b*, which were highly upregulated after rat ONT, but not after mouse ONC. *Casp4* (also known as *Casp11*) is a part of the caspase family and plays an essential role in apoptosis^42^. CASP11 protein has been reported to be involved in the activation of the inflammatory response during injuries to the central nervous system^43^, and has previously been found to be upregulated in rat retinas after ONT^44^. CASP11 can promote the processing of caspase-3, leading to cell death^45^. Therefore, CASP11 may play an important role in RGC death after ONT in rats. Another highly upregulated DEG found only in the rat ONT model was *Ifi27l2b*, also known as *ISG12b2*^46^. ISG12b2 protein induces the release of cytochrome c from the mitochondria, triggering mitochondria-mediated apoptosis^46^. Yang *et al*. used a microarray analysis to compare DEGs in rat retinas in two models: one using ONT and another using artificial IOP elevation^34^; they found that *ISG12b2* was upregulated in both models. However, the upregulation began sooner after ONT than it did after IOP elevation. Axonal damage was immediate and severe after ONT^12^, whereas it occurred only slowly and was moderate after IOP elevation. This suggests that *ISG12b2* upregulation may have an association with the extent of axonal damage.

RNA-seq analysis has important advantages over previous methods based on microarray analysis, which were reliant on hybridization probes of known sequences. RNA-seq can detect unknown transcripts and estimate the expression level of different transcript isoforms6. In this study, we identified 49 unannotated DEGs after ONT, which may include novel transcripts, and which may be involved in the pathomechanisms of axonal injury. Moreover, we identified hundreds of differentially expressed transcripts, including potentially novel splice isoforms. Isoforms of the same gene may contribute to the production of protein variants and add to the diversity of cellular function under a variety of biological conditions^47^. One recent example was the finding that isoform-level expression changes were involved in the progression of Alzheimer’s disease^48^. RNA-seq and the Cufflinks analysis software allowed us to identify many transcripts that were differentially expressed after ONT, including 103 potentially novel isoforms. *Chac1* was among the highly upregulated transcripts that the Cufflinks software identified as having potentially novel isoforms. The elevated mRNA expression of *Chac1* splice isoforms has been reported to be associated with poor prognosis in human breast and ovarian cancer^49^. *Chac1* isoform level expression changes might also be involved in unexplored pathomechanisms of axonal injury. However, we have not validated these expression changes in splice isoforms, and lack information on their functional impact. Further analysis will be necessary to determine their role in the pathomechanisms of axonal injury.

This study also included an *in silico* functional analysis of the DEGs based on IPA, which revealed that “cell-to-cell signaling and interaction” was the most significant molecular and cellular function after ONT. *Tnfrsf12a* (also known as the TWEAK receptor^50^) was one of the most highly upregulated DEGs in our RNA-seq analysis and belonged to both the “cell-to-cell signaling and interaction” and “cell death and survival” functions. *Tnfrsf12a* has been reported to be upregulated in several different models of axonal injury^8^,^10^,^11^. However, the functional association of this gene with the pathology of axonal injury remains unclear. *Tnfrsf12a* is involved in multiple biological activities, including inflammation and apoptosis. Bhattacharjee M *et al*. contributed to the understanding of this gene by developing a bioinformatics database for the TWEAK-Fn14 signaling pathway^51^. According to this database, *Tnfrsf12a* is involved in NFKB^52^, MAPK^53^ and JNK^54^, all of which have been reported to play important roles in axonal injury. In combination with our findings, this suggests that *Tnfrsf12a* may be a key mediator in axonal injury-driven RGC death.

We used IPA to perform an upstream regulator analysis of the DEGs, in order to determine the most important transcription factors governing pathological networks in the retina after axonal injury. According to this analysis, the most significant upstream transcription regulator after ONT was CREB1, also known as CREB. This transcription regulator is activated in response to harmful stress stimuli, such as hypoxia and oxidative stress, and is involved in the cellular defense against these stresses^55^. CREB has been reported to promote neuronal survival^56^, and a recent study showed that CREB has a neuroprotective effect against hydrogen peroxide-induced RGC death via two cell survival genes downstream of it: *Bdnf* and *Bcl-2*^57^. Here, IPA showed that CREB1 targeted a number of DEGs, including the following cytoprotective genes: *Ecel1*, *Dusp1*^58^, *Gpmnb*, *Hmox1*, *Srxn1*^59^, and *Vgf *60. Thus, the CREB1 cascade may play an important part in the neuroprotective response to axonal injury.

The IPA upstream regulator analysis predicted that ATF4 and DDIT3 were significant upstream regulators after ONT. Previously, we found that ATF4 and DDIT3 were also significant upstream regulators in a mouse ONC model^10^, a finding that was consistent with the current study. The ATF4-DDIT3 pathway is activated under ER stress, inducing cell death^28^, and DDIT3 (also known as CHOP) has been reported to be associated with RGC death after mouse ONC^61^. These findings suggest that the ATF4-DDIT3 pathway plays a key role in axonal injury-driven RGC death. Interestingly, we found that ATF4 included *Tnfrsf12a* as one of its downstream target molecules (Table 3). As described above, *Tnfrsf12a* has been reported to be involved in inflammation and apoptosis. However, a search of the relevant literature reveals no investigations into the involvement of the ATF4-*Tnfrsf12a* pathway in axonal injury, although it has been reported that ATF4 induces Toll-like receptor 2 (TLR2) upregulation under ER stress and under oxidative stress, leading to neuroinflammation^62^. It has also been reported that TLR2 is involved in *Tnfrsf12a* upregulation in herpes simplex virus-infected microglia, leading to apoptotic cell death^63^. Together, these findings suggest that the ATF4-*Tnfrsf12a* pathway, activated by ER stress, induces neuroinflammation and contributes to axonal injury-driven RGC death.

IPA also predicted that NUPR1 (nuclear protein 1) was an upstream transcription regulator involved in molecular changes after ONT in rats. NUPR1 is a protein that has previously been reported to be overexpressed in pancreatic acinar cells during the acute phase of pancreatitis^64^. However, it is unknown how NUPR1 may undergo expression changes in the RGCs or how these changes contribute to RGC death after axonal injury. *Nupr1* expression is regulated by ATF4, which is induced by diverse cellular stresses, including ER stress and oxidative stress^29^,^65^. NUPR1 is involved in various cellular functions, including apoptosis and autophagy^66^. In addition, NUPR1 has been reported to cause Akt-mediated phosphorylation and subsequent cytoplasmic re-localization of p21, which leads to activation of the anti-apoptotic Bcl-xL protein^29^,^67^. We thus propose NUPR1 as a novel therapeutic target for the prevention of RGC death after axonal injury.

In this study, we performed RNA-seq and an *in silico* pathway analysis of whole retinal samples, but not purified samples of RGCs. Thus, the gene expression changes identified in this study may represent other types of retinal cell (e.g., the microglia), in addition to the RGCs. Nevertheless, we consider that this method was valid, because the process of dissociating and purifying the RGCs can itself damage the cells and induce additional gene expression changes. In addition, though glial cell activity plays an important role in axonal injury-driven RGC death, such changes in gene expression do not occur in purified RGC samples. We thus consider that the use of complete retinal samples in this study was the most suitable way of showing the overall response of the retina to axonal injury, and believe that our study should provide a useful database for an improved understanding of the pathomechanisms of axonal injury.

## Conclusions

In summary, we used RNA-seq and *in silico* pathway analysis to identify a number of DEGs (including potentially novel isoforms) and upstream regulators, that may be involved in axonal injury in the rat retina after ONT. Among these, *Ecel1, Tnfrsf12a, Gpnmb, Chac1*, CREB1 and NUPR1 were the most promising candidates for the development of novel neuroprotection strategies against axonal injury-driven RGC death. Although it is still necessary to perform a functional analysis of these DEGs and their upstream transcription regulators, we believe that this study constitutes a valuable new resource for research on axonal injury-driven RGC death, and that it promises to aid in drug discovery for new neuroprotective treatments for glaucoma.

## Methods

### Animals

Eighteen Sprague-Dawley rats (male, 8 weeks old; SLC, Hamamatsu, Japan) were used in this study. All animals were maintained and handled in accordance with the ARVO Statement for the Use of Animals in Ophthalmic and Vision Research and the Declaration of Helsinki. The Ethics Committee for Animal Experiments at Tohoku University Graduate School of Medicine approved all experimental procedures described in the present study.

### Axonal injury surgical procedure

Axonal injury was induced by transecting the right optic nerve, as previously described^31^,^32^. Briefly, after anesthetization with an intraperitoneal injection of sodium pentobarbital (Nembutal; 45 mg/kg), the eye was drawn down, the upper conjunctiva was incised, and the optic nerve was exposed. The sheath of the optic nerve was then cut longitudinally, and the optic nerve was transected about 1 mm posterior to the eyeball, with care taken not to damage the local vessels. Sham operations were also performed on a separate group of rats. In the sham operation, the optic nerve was not transected but the procedure was otherwise identical.

### RNA preparation

Total RNA was purified from each retinal sample as previously described^10^. Briefly, the retinas were extracted 2 days after surgery, and immediately immersed in RNAlater RNA Stabilization Reagent (Qiagen, Tokyo, Japan). The retinas were then homogenized in Qiazol (Qiagen) with a pestle homogenizer, and total RNA was extracted from the homogenized mixture with a miRNeasy mini kit (Qiagen). The total RNA concentration and quality were evaluated with a spectrophotometer (NanoDrop 2000c, Thermo Scientific). Next, the expression levels of 5 typical RGC markers (*Pou4f1*, *Pou4f2*, *Pou4f3*, *Nefl*, and *Thy1*)^68^,^69^ were evaluated with qRT-PCR, to confirm that the RGCs were uninjured in the sham group and injured in the ONT group. To prepare the RNA samples for RNA-seq, 5 μg of RNA were taken from three samples and combined into a single sample, in order to minimize the influence of individual variations in the rats^10^,^11^. The quality of these resulting six RNA samples (each containing RNA from 3 animals) was then assessed with an Agilent 2100 Bioanalyzer (Agilent Technologies). The RNA integrity number of each sample used for the preparation of the cDNA library for RNA-seq is shown in Table S1.

### cDNA library preparation and RNA sequencing

We used a Truseq RNA sample prep kit v2 (Illumina) to prepare a cDNA library for RNA-seq, following the manufacturer’s protocol. A total of 1.5 μg of RNA from each retinal sample was used to synthesize the cDNA library, which was used for 100 bp paired-end sequencing. We prepared six cDNA libraries (in triplicate) for cluster generation and sequencing. Each of them was indexed to allow for multiplex sequencing, and then applied to an Illumina flow cell for cluster generation. Clonal clusters were generated using TruSeq PE Cluster Kit v3-cBot-HS (Illumina) on a cBot fluidics device according to the manufacturer’s protocol. After cluster generation, sequencing was performed on one lane of the Illumina Hiseq2500 device. The resulting sequence data were recorded in the FASTQ format. All RNA-seq data are available under the accession number DRA004746.

### Sequence data processing and differential expression analysis

For RNA-seq data analysis, we used an integrated pipeline provided by the Data Analysis Center of the Cell Innovation Program (http://cell-innovation.nig.ac.jp). The pipeline consists of Tophat, Cufflinks^22^, and CummeRbund^21^ software.

The RNA-seq reads in FASTQ format were imported into the pipeline, and then aligned to a reference genome (rn4) with Tophat (v2.0.6) resulting in BAM format files. The resulting aligned reads were analyzed further with Cufflinks (v2.0.2). Cufflinks was also used to assemble transcripts from RNA-seq reads and estimate their abundance as FPKM^20^, which normalizes transcript expression for transcript length and the total number of reads per sample. Cuffdiff, a part of the Cufflinks package, was used to identify DEGs and transcripts in the ONT and sham groups. *P*-values were adjusted for multiplicity with Benjamini-Hochberg correction^70^, and *q*-values were calculated. Genes and transcripts with *q*-values <0.05 and absolute fold-change >1.5 were considered differentially expressed. We used The CummeRbund software to visualize the RNA-seq data produced by Cufflinks.

### QRT-PCR

Eighteen samples of purified RNA (n = 9 in each group) were examined with qRT-PCR. Total RNA (500 ng per sample) from each sample was reverse-transcribed into cDNA using SuperScript III (Invitrogen Life Technologies). QRT-PCR was then performed with a 7500 Fast Real-Time PCR System (Applied Biosystems, Foster City, CA) as previously described^10^. For each 20 μl reaction the following were used: 10 μl TaqMan Fast Universal PCR Master Mix (Applied Biosystems), 1 μl Taqman probe, 1 μl template DNA, and 8 μl DEPC water. All qRT-PCR assays were performed in duplicate. For a relative comparison of gene expression, we analyzed the results of the qRT-PCR data with the comparative Ct method (2^- ddCT^)^19^, normalized to *Gapdh*, an endogenous control. The TaqMan probes used for these reactions were *rCG37080*: Rn01753495_m1 (after the start of this study, this probe was deprecated for use as a primer for *Pou4f1*); *Pou4f1*: Rn01465571_m1; *Pou4f2*: Rn01431271_g1; *Pou4f3*: Rn00454761_g1; *Nefl:* Rn00582365_m1, *Thy1*: Rn00562048_m1; *Casp4*: Rn00586960_m1; *Chac1*: Rn01747607_m1; *Ecel1*: Rn00572963_m1; *Ifi27l2b*: Rn01517868_g1; *Lgals3*: Rn00582910_m1; *Rbpms2*: Rn01422469_m1; *Stc2*: Rn00573702_m1; *Tnfrsf12a*: Rn00710373_m1; *Gapdh*: Rn01462662_g1. Initially, this study used the TaqMan probe Rn01753495_m1 as a primer for *Pou4f1*, but the manufacturer subsequently deprecated this probe because the reference sequence (RefSeq) of Rn01753495_m1 (XM 341372.4) was removed from the NCBI GenBank (http://www.ncbi.nlm.nih.gov/nuccore/XM_341372.4?report=genbank). Thus, in subsequent analyses, this primer was replaced with the *Pou4f1*: Rn01465571_m1 probe.

### *In silico* functional and pathway analysis

To identify how the observed gene expression changes in the groups altered biological processes and signaling pathways, we performed an *in silico* functional and pathway analysis with IPA (Ingenuity Systems, Redwood City, CA), as previously described^11^. Briefly, the datasets of the DEGs after ONT were uploaded to the IPA application and mapped to the Ingenuity Pathways Knowledge Base. The datasets and biofunctions were measured as the ratio of the number of genes from the dataset that mapped to the pathway divided by the total number of genes in that pathway. In order to investigate the regulatory networks after ONT, we performed an upstream regulator analysis to compare DEGs in the datasets to those known to be regulated by a given upstream regulator^11^,^71^. Based on the concordance between them, we assigned an activation score, showing whether a potential upstream regulator was in an “activated” (z-score ≥ 2), “inhibited” (z-score ≤ −2), or uncertain state.

### Statistical analysis

Statistical analysis of the qRT-PCR data was performed with R software^72^ (version 3.1.1). QRT-PCR data were analyzed with Welch’s *t*-test. The significance of the pathway analysis was calculated with Fisher’s exact test in the IPA application. If the *P*-values for qRT-PCR and IPA were less than 0.05, the results were considered statistically significant.

## Additional Information

**How to cite this article**: Yasuda, M. *et al*. Transcriptome profiling of the rat retina after optic nerve transection. *Sci. Rep*. **6**, 28736; doi: 10.1038/srep28736 (2016).

## Supplementary Material

Supplementary Information

## Figures and Tables

**Table 1:** Top 20 upregulated genes after ONT.

Symbol	Description	RefSeq	Log_2_ FC	q-value
Up-regulated				
Ecel1*,†	Endothelin converting enzyme-like 1	NM_021776	5.86	1.60E-10
*Casp4*†	Caspase 4, apoptosis-related cysteine peptidase	NM_053736	4.54	2.61E-05
*Stc2*	Stanniocalcin 2	NM_022230	4.07	6.39E-14
*Ifi27l2b*	Interferon, alpha-inducible protein 27 like 2B	NM_206846	3.76	0.00E + 00
Tnfrsf12a*	Tumor necrosis factor receptor superfamily, member 12a	NM_181086	3.68	0.00E + 00
*Mmp19*	Matrix metallopeptidase 19	NM_001107159	3.63	7.89E-03
*Flnc*	Filamin C, gamma	NM_001191862	3.35	0.00E + 00
Gpnmb*	Glycoprotein (transmembrane) nmb	NM_133298	3.32	0.00E + 00
Lgals3*	Lectin, galactoside-binding, soluble, 3	NM_031832	2.93	9.05E-08
*Krt12*	Keratin 12	NM_001008761	2.89	7.62E-06
*Hspb1*†	Heat shock 27kDa protein 1	NM_031970	2.74	4.18E-13
*Arc*	Activity-regulated cytoskeleton-associated protein	NM_019361	2.71	1.34E-07
Hmox1*	Heme oxygenase 1	NM_012580	2.65	5.30E-13
Csrnp1*	Cysteine-serine-rich nuclear protein 1	NM_001108786	2.63	8.75E-08
*Lcn2*	Lipocalin 2	NM_130741	2.52	6.80E-12
*RGD1564664*	Similar to LOC387763 protein	NM_001110055	2.43	0.00E + 00
*Csrp3*†	Cysteine and glycine-rich protein 3	NM_057144	2.42	1.22E-08
*Creb5*	cAMP responsive element binding protein 5	NM_001134621	2.39	3.93E-02
Sox11*	SRY (sex determining region Y)-box 11	NM_053349	2.36	0.00E + 00
Chac1*,†	ChaC, cation transport regulator 1	NM_001173437	2.34	0.00E + 00

Differences were considered significant when the q-value was <0.05 and |FC| was >1.5.

^*^Overlapping upregulated genes in the rat ONT and mouse ONC models.

^†^DEGs with potentially novel isoform.

**Table 2:** Top 5 molecular and cellular functions altered after ONT.

Category	*P*-value	Number of molecules
Cell-to-cell signaling and interaction	1.55E-10 - 1.07E-02	73
Cell death and survival	1.32E-09 - 1.08E-02	80
Cellular development	1.35E-09 - 9.72E-03	82
Cell morphology	1.68E-09 - 1.01E-02	71
Cellular assembly and organization	1.68E-09 - 1.01E-02	55

Significances were calculated with Fisher’s exact test. Differences were considered significant at *P* <0.05.

**Table 3:** Upstream transcription regulators predicted to be altered after ONT.

Upstream Regulator	Activation z-score	*P*-value of overlap	Target molecules in dataset
CREB1	3.69	1.71E-12	ARC, ATF3, ATF5, CALB2, CARS, CDKN1A, CSRNP1, DUSP1, ECEL1, EGR1, GADD45G, GPNMB, HMOX1, HTR2A, INA, JUN, KCND2, LCN2, LPPR1, NOS1, Pvr, RPRM, SERINC2, SLC17A6, SPOCK3, SRXN1, TAC1, VGF
ATF4	3.06	4.18E-11	ASNS, ATF3, ATF5, CDKN1A, CTH, EIF4EBP1, JUN, LGALS3, MTHFD2, PHGDH, SLC7A1, SLC7A3, STC2, TNFRSF12A
STAT3	2.55	1.57E-03	CCND2, CDKN1A, CHI3L1, EGR1, FLRT3, GADD45G, GFAP, HMOX1, HTR2A, PIM1, SHH, STC2
RELA	2.40	8.82E-03	ABCB1, CDKN1A, CHI3L1, DUSP1, EGR1, HMOX1, JUN, SHH, TAC1
DDIT3	2.39	4.89E-06	ATF3, ATF5, CASP4, HMOX1, LCN2, MLF1
SMAD4	2.22	6.45E-02	CCND2, CDKN1A, HMOX1, IRAK3, SHH
SP1	2.21	2.33E-04	ABCB1, ASNS, ATF3, CCND2, CDKN1A, CHI3L1, CHRNA3, CTH, EGR1, HMOX1, JUN, NOS1, PIM1, SLC7A1, SNCG
PDX1	2.20	5.67E-04	ANXA2, ATF3, EGR1, GFAP, JUN, KLF6, PHGDH, PPP2R2C
SMAD3	2.19	7.39E-02	CCND2, CDKN1A, EGR1, HMOX1, JUN
EGR1	2.13	7.31E-05	ABCB1, ARC, ATF3, CCND2, CDKN1A, EGR1, EIF4EBP1, HMOX1, JUN
NUPR1	2.00	1.00E-00	ATF3, CREB5, KLF6, PIM1
NFYA	2.00	8.86E-03	ABCB1, CDKN1A, EGR1, JUN
