# Supplementary material for: Transcriptome profiling of the rat retina after optic nerve transection
Source: Sci Rep. 2016 Jun 29;6:28736. doi: 10.1038/srep28736 (PMC4926057; doi:10.1038/srep28736)
Supplement: Supplementary Information [file srep28736-s1.pdf]

Supplementary information

Transcriptome profiling of the rat retina after optic nerve transection

Masayuki Yasuda[1], Yuji Tanaka[2], Kazuko Omodaka[1], Koji M Nishiguchi[3], Orie Nakamura[1], Satoru Tsuda[1], Toru Nakazawa[1,3,4]

1. Department of Ophthalmology, Tohoku University Graduate School of Medicine, 1-1 Seiryo-machi, Aoba-ku, Sendai, Miyagi, 980-8574, Japan.
2. RIKEN Center for Life Science Technologies (Division of Genomic Technologies), 1-7-22 Suehiro-cho, Tsurumi-ku, Yokohama, Kanagawa 230-0045, Japan.
3. Department of Advanced Ophthalmic Medicine, Tohoku University Graduate School of Medicine, 1-1 Seiryo-machi, Aoba-ku, Sendai, Miyagi, 980-8574, Japan.
4. Department of Retinal Disease Control, Tohoku University Graduate School of Medicine, 1-1 Seiryo-machi, Aoba-ku, Sendai, Miyagi, 980-8574, Japan.

Corresponding author:

Toru Nakazawa, MD, PhD

Tohoku University Graduate School of Medicine, Department of Ophthalmology, 1-1 Seiryo, Aoba, Sendai, Miyagi, Japan

Zip code: 980-8574, Phone: +81-22-717-7294, Fax: +81-22-717-7298

E-mail: ntoru@oph.med.tohoku.ac.jp

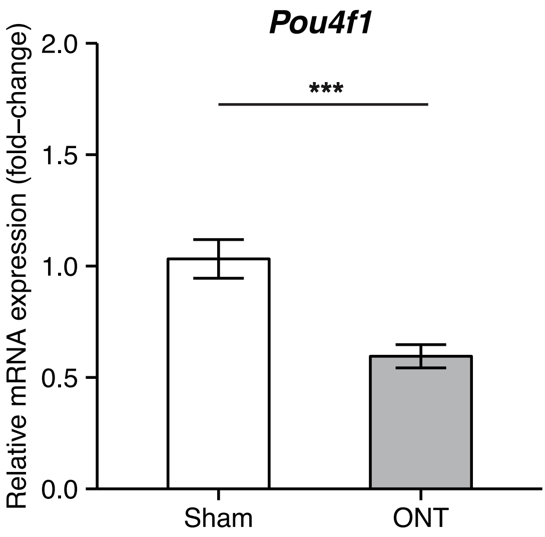

Supplementary Figure S1. Revalidation of *Pou4f1* mRNA expression changes after ONT with qRT-PCR and updated probe

The *Pou4f1* mRNA expression level in each sample (n = 9 in each group) used to prepare the cDNA library was revalidated with qRT-PCR and the updated TaqMan probe for *Pou4f1* (ID: Rn01465571_m1). The graph shows mRNA expression in the ONT and sham groups, normalized to an average expression of 1.0 in the sham group. Values are mean ± S.E.M. (***$P < 0.001$). *Pou4f1* mRNA expression was significantly downregulated after ONT.

Table S1. The typical mRNA expression of the RGC markers and the RNA quality of each sample

Sample number	Sequence sample name	260/280	260/230	Ct*Gapdh*	dCt*Pou4f1*	dCt*Pou4f2*	dCt*Pou4f3*	dCt*Nefl*	dCt*Thy1*	ddCt*Pou4f1*	ddCt*Pou4f2*	ddCt*Pou4f3*	ddCt*Nefl*	ddCt*Thy1*	ddCtRGC	Mean ddCtRGC	Mean - 3SD of ddCtRGC	Mean + 3SD of ddCtRGC
1		2.07	2.04	20.156	13.979	8.971	12.573	3.151	4.233	0.025	0.025	0.259	-0.198	0.014	0.126			
2	Sham1	2.04	2.04	20.210	13.989	9.271	12.278	3.278	4.299	0.035	0.325	-0.036	-0.071	0.080	0.333			
3		2.06	2.10	20.348	14.246	9.314	12.343	3.714	4.432	0.292	0.367	0.029	0.365	0.213	1.266			
4		2.06	2.04	20.638	13.642	8.516	12.034	4.006	3.887	-0.312	-0.431	-0.280	0.657	-0.332	-0.697			
5	Sham2	2.04	2.07	20.317	13.741	8.969	12.366	3.100	4.091	-0.213	0.022	0.052	-0.249	-0.128	-0.516	0.000	-1.994	1.994
6		2.04	1.99	20.281	14.235	8.787	12.182	3.267	4.055	0.281	-0.159	-0.132	-0.082	-0.164	-0.255			
7		2.06	1.97	20.075	13.937	9.165	12.478	3.266	4.279	-0.017	0.219	0.164	-0.083	0.060	0.343			
8	Sham3	2.05	2.06	20.147	13.602	8.654	12.280	3.128	4.231	-0.352	-0.293	-0.033	-0.221	0.012	-0.887			
9		2.06	1.94	20.237	14.216	8.870	12.292	3.230	4.462	0.262	-0.076	-0.022	-0.119	0.243	0.288			
10		2.08	2.03	21.046	17.320	9.417	12.484	4.259	5.132	3.366	0.471	0.170	0.910	0.913	5.831			
11	ONT1	2.07	1.91	20.683	16.247	10.010	12.647	4.112	5.254	2.293	1.063	0.333	0.763	1.035	5.488			
12		2.07	1.84	20.786	17.076	10.701	12.783	4.325	5.347	3.122	1.755	0.469	0.976	1.128	7.449			
13		2.08	2.05	20.691	16.371	10.288	12.823	4.227	5.339	2.417	1.341	0.509	0.878	1.120	6.266			
14	ONT2	2.07	2.07	20.623	15.770	10.038	12.567	3.908	4.862	1.816	1.092	0.253	0.559	0.643	4.363	5.696	2.364	9.028
15		2.06	2.05	20.429	16.089	10.219	12.522	4.257	5.432	2.135	1.272	0.208	0.908	1.213	5.736			
16		2.06	2.02	20.432	16.500	10.615	12.985	4.216	5.487	2.546	1.669	0.671	0.867	1.268	7.020			
17	ONT3	2.08	1.78	20.543	15.934	9.581	12.565	4.014	4.874	1.980	0.634	0.251	0.665	0.655	4.186			
18		2.06	2.06	20.579	15.857	10.530	12.610	3.796	4.913	1.903	1.583	0.296	0.447	0.694	4.923			

dCt: *Gapdh* normalized Ct value. ddCT was calculated using the following formula: ddCt = dCt - mean dCT of the sham group. ddCtRGC: Sum of ddCt of the 5 RGC markers (*Pou4f1*, *Pou4f2*, *Pou4f3*, *Nefl*, and *Thy1*). SD: Standard deviation.

Table S2. RNA integrity numbers and summary of sequence statistics

Sample	RIN	Total number of reads	Total number of mapped reads	Mapping rate (%)
Sham1	8.8	77,303,084	62,845,013	81.3
Sham2	9.2	56,134,578	46,587,355	83.0
Sham3	9.2	58,125,310	47,767,732	82.2
ONT1	9.1	65,379,406	53,309,235	81.5
ONT2	8.7	63,056,578	50,931,036	80.8
ONT3	8.8	62,013,204	50,300,518	81.1

RIN: RNA integrity numbers

Table S3. List of DEGs after ONT

Symbol	RefSeq	Gene locus	log_2FC	q-value
Ecel1	NM_021776	chr9:85933919-85936173	5.86	1.60E-10
Casp4	NM_053736	chr8:2038574-2076531	4.54	2.61E-05
-	NA	chr16:26443686-26444348	4.23	1.39E-11
Stc2	NM_022230	chr10:16514554-16526844	4.07	6.39E-14
Ifi27l2b	NM_206846	chr6:127733546-127735079	3.76	0.00E+00
-	NA	chr8:73982152-74010606	3.69	8.92E-12
Tnfrsf12a	NM_181086	chr10:12940402-12942373	3.68	0.00E+00
Mmp19	NM_001107159	chr7:2091344-2099717	3.63	7.89E-03
Flnc	NM_001191862	chr4:56313604-56341381	3.35	0.00E+00
Gpnmb	NM_133298	chr4:77161283-77182621	3.32	0.00E+00
Lgals3	NM_031832	chr15:23326768-23339109	2.93	9.05E-08
Krt12	NM_001008761	chr10:88320793-88328809	2.89	7.62E-06
Hspb1	NM_031970	chr12:21911202-21912866	2.74	4.18E-13
Arc	NM_019361	chr7:112771934-112775365	2.71	1.34E-07
Hmox1	NM_012580	chr19:13963009-13969949	2.65	5.30E-13
Csrnp1	NM_001108786	chr8:124933482-124945923	2.63	8.75E-08
Lcn2	NM_130741	chr3:11511400-11515051	2.52	6.80E-12
RGD1564664	NM_001110055	chr3:78365767-78372059	2.43	0.00E+00
Csrp3	NM_057144	chr1:98601687-98620255	2.42	1.22E-08
Creb5	NM_001134621	chr4:81616078-81617489	2.39	3.93E-02
Sox11	NM_053349	chr6:45135451-45143452	2.36	0.00E+00
Chac1	NM_001173437	chr3:105864667-105873323	2.34	0.00E+00
Krt13	NM_001004021	chr10:89057009-89061202	2.20	1.71E-03
-	NA	chr3:40019207-40022593	2.16	2.12E-03
Atf3	NM_012912	chr13:107191621-107224007	2.15	0.00E+00
Ipcef1	NM_001170799	chr1:37631404-37711075	2.08	0.00E+00
Cpm	NM_001108098	chr7:56932156-56992185	2.02	2.53E-05
-	NA	chr16:60118901-60136536	1.99	1.33E-02
Egr1	NM_012551	chr18:27343329-27347353	1.94	0.00E+00
Serinc2	NM_001031656	chr5:149279202-149301822	1.93	4.42E-03
Agpat9	NM_001025670	chr14:9997584-10051174	1.85	2.19E-03
-	NA	chr14:80956641-80960508	1.82	1.30E-02
Il28ra	NM_001191868	chr5:154461480-154483067	1.79	2.32E-03
Cd24	NM_012752	chr20:47492883-47507659	1.65	2.32E-05
Mir431	NR_031953	chr6:134181230-134186527	1.64	1.92E-05
Anxa1	NM_012904	chr1:223478450-223494553	1.60	3.78E-03
-	NA	chr16:9815114-9831562	1.59	1.87E-03
Arid5a	NM_001034934	chr9:35231518-35246491	1.56	1.87E-11
Pim1	NM_017034	chr20:7817008-7822801	1.52	5.57E-09
Irak3	NM_001108101	chr7:59407094-59468168	1.46	4.11E-03
-	NA	chr4:171706666-171731351	1.46	3.57E-04

RGD1566319	NM_001109358	chr5:152639880-152655830	1.45	5.60E-04
Eif4ebp1	NM_053857	chr16:69110555-69123897	1.43	2.05E-02
-	NA	chr1:1588230-1594890	1.41	1.49E-03
Stk32a	NM_001191894	chr18:36501670-36614795	1.40	0.00E+00
Slc7a1	NM_013111	chr12:7230392-7253853	1.33	0.00E+00
Shc4	NM_001191065	chr3:112936087-113030536	1.29	2.39E-02
Stmn4	NM_019176	chr15:45779151-45797534	1.29	0.00E+00
Asns	NM_013079	chr4:32753093-32776628	1.25	0.00E+00
PVR	NM_017076	chr1:79212729-79229069	1.21	2.54E-04
Srxn1	NM_001047858	chr3:142457296-142463397	1.21	0.00E+00
Nos1	NM_052799	chr12:39811703-39893785	1.18	2.45E-08
-	NA	chr9:85988788-85993978	1.17	0.00E+00
C1qtnf1	NM_001007675	chr10:108428236-108453209	1.13	3.51E-03
-	NA	chr7:57333751-57337699	1.11	4.28E-04
-	NA	chr7:57340416-57343593	1.10	4.32E-02
-	NA	chr20:3961364-3964119	1.09	2.40E-09
Vgf	NM_030997	chr12:20898444-20906638	1.07	0.00E+00
Hspb8	NM_053612	chr12:41407000-41421553	1.07	1.31E-10
Ramp3	NM_020100	chr14:87417276-87434785	1.06	1.11E-02
-	NA	chr5:157619679-157649295	1.05	3.85E-02
Stim2	NM_001105750	chr14:61631936-61759973	1.03	5.57E-04
Cyp4v3	NM_001135600	chr16:50208106-50233722	1.02	3.41E-03
C2cd4c	NM_001109285	chr7:11608875-11612166	1.01	6.67E-03
Hs3st5	NM_001106392	chr20:40711674-41022763	1.01	8.12E-04
Cth	NM_017074	chr2:256056567-256082450	1.00	2.23E-02
-	NA	chr7:26147257-26154852	1.00	2.99E-02
Mlf1	NM_001107680	chr2:157230756-157263924	0.99	5.24E-03
Kcnk12	NM_022292	chr6:11277526-11355065	0.98	1.43E-08
Lonrf3	NM_001191585	chrX:8562796-8566005	0.97	5.55E-03
Fam129a	NM_022242	chr13:66467068-66621851	0.97	5.80E-04
Gadd45g	NM_001077640	chr17:19230900-19232842	0.97	1.24E-13
Mthfd2	NM_001109398	chr4:117517605-117529130	0.95	1.34E-12
Tac1	NM_012666	chr4:32648884-32657616	0.94	7.76E-10
RGD1359634	NM_001007708	chr7:117087031-117122728	0.94	3.42E-05
Wls	NM_199408	chr2:258014378-258128850	0.94	9.63E-07
Sdcbp	NM_031986	chr5:19822305-19849244	0.93	0.00E+00
Ccnd2	NM_022267	chr4:163519302-163546744	0.93	3.01E-12
Rab15	NM_198749	chr6:99378936-99403488	0.92	0.00E+00
Atf5	NM_172336	chr1:95284083-95303547	0.90	0.00E+00
Pla2g5	NM_017174	chr5:157619679-157649295	0.90	5.28E-03
Slc7a3	NM_017217	chrX:89159609-89165672	0.90	2.99E-10
Slc6a8	NM_017348	chrX:159570631-159580110	0.88	0.00E+00
Bspry	NM_022261	chr5:79471064-79498789	0.87	2.56E-03

-	NA	chr1:208476329-208480254	0.87	1.29E-02
Anxa2	NM_019905	chr8:73897548-73934051	0.84	2.01E-11
-	NA	chr17:26284045-26291438	0.84	0.00E+00
Clic1	NM_001002807	chr20:3498999-3499328	0.82	3.98E-02
Abcb1a	NM_133401	chr4:21497686-21796596	0.82	1.31E-03
-	NA	chr4:163620377-163642092	0.81	2.01E-11
Rhoq	NM_053522	chr6:10414284-10474868	0.80	9.93E-06
Klf6	NM_031642	chr17:75576214-75585151	0.79	5.28E-10
Phgdh	NM_031620	chr2:193147938-193177099	0.78	1.61E-07
Cdkn1a	NM_080782	chr20:7385786-7386778	0.78	1.26E-02
Gfap	NM_017009	chr10:92059871-92068795	0.77	0.00E+00
Eno4	NM_001134505	chr1:265440185-265491907	0.76	1.53E-03
Aldh18a1	NM_001108524	chr1:245685968-245718297	0.74	2.34E-09
-	NA	chr1:208476329-208480254	0.74	6.40E-13
Mafk	NM_145673	chr12:15312413-15323194	0.73	1.42E-02
Vwa5a	NM_198755	chr8:40850216-40875406	0.73	2.76E-02
Slc1a4	NM_198763	chr14:101122507-101161667	0.72	8.40E-11
Setd7	NM_001109558	chr2:140408843-140457440	0.71	0.00E+00
Jun	NM_021835	chr5:115358175-115378138	0.70	0.00E+00
RGD1311249	NM_001013931	chr5:59523094-59536867	0.69	1.13E-02
Htr2a	NM_017254	chr15:55462700-55535175	0.69	2.46E-03
Flrt3	NM_001126291	chr3:128921577-128935096	0.68	3.70E-07
-	NA	chr10:35904933-35910391	0.68	7.46E-03
Cars	NM_001106319	chr1:203934719-203977051	0.67	2.47E-10
Dusp1	NM_053769	chr10:16942654-16945693	0.66	4.86E-08
Tnik	NM_001106422	chr2:115597332-116009522	0.66	4.74E-11
Tes	NM_001039344	chr4:42680546-42719249	0.65	7.94E-04
Chi3l1	NM_053560	chr13:47135405-47147631	0.65	4.02E-08
Gpr137c	NM_001134541	chr15:21029771-21167044	0.64	4.52E-04
Plekho1	NM_001025119	chr2:190787512-190796471	0.62	2.23E-03
-	NA	chr1:186318573-186329889	0.60	6.45E-09
Eef1a2	NM_012660	chr3:170295678-170307673	0.60	0.00E+00
Adam23	NM_001029899	chr9:62105884-62253219	-0.59	5.60E-04
Pcdh8	NM_022868	chr15:61056318-61063949	-0.59	2.18E-04
Grik3	NM_181373	chr5:144842414-145063315	-0.59	1.05E-02
Adam11	NM_001108300	chr10:91880528-91899987	-0.59	4.30E-05
Pcdh7	NM_001004087	chr14:57257427-57326354	-0.59	1.13E-05
Sorl1	NM_053519	chr8:44958443-45101939	-0.59	0.00E+00
Necab2	NM_133415	chr19:49691464-49718720	-0.60	1.71E-03
Mapk13	NM_019231	chr20:7055294-7065597	-0.60	3.64E-02
Rbfox1	NM_001106974	chr10:8213471-8601179	-0.60	2.40E-06
Scn2a1	NM_012647	chr3:47501904-47723248	-0.61	6.59E-07
Matk	NM_021859	chr7:9936608-9946664	-0.61	9.50E-04

Slc35f1	NM_001109338	chr20:31209336-31770161	-0.61	2.89E-09
-	NA	chr14:44360756-44527771	-0.62	1.05E-03
Atl1	NM_001009831	chr6:91884703-92077631	-0.62	2.75E-06
-	NA	chr5:61982036-62090680	-0.63	4.21E-04
Snca	NM_019169	chr4:89613822-89723335	-0.63	0.00E+00
Ywhah	NM_013052	chr14:83448397-83457891	-0.63	0.00E+00
Bend6	NM_001108792	chr9:32607617-32656101	-0.63	6.49E-05
Rnf148	NM_001191082	chr4:49658565-50206224	-0.64	3.37E-02
Magee1	NM_001079891	chrX:93191779-93195782	-0.64	1.11E-11
Fdps	NM_031840	chr2:181166191-181168360	-0.64	6.43E-04
Vwa5b2	NM_001134535	chr11:82529324-82552952	-0.65	3.04E-02
-	NA	chrX:52695146-53089190	-0.65	9.16E-06
Cacng5	NM_080693	chr10:97297843-97342782	-0.66	4.23E-06
Mmp15	NM_001106168	chr19:10122504-10143923	-0.66	1.63E-03
Oprl1	NM_031569	chr3:170869118-170880725	-0.66	8.84E-03
Fgf12	NM_130814	chr11:74098767-74486322	-0.66	1.24E-07
-	NA	chr11:41262609-41470453	-0.66	4.12E-04
Spock3	NM_001107310	chr16:29751354-29751958	-0.67	2.07E-02
Nptx1	NM_153735	chr10:108918366-108927773	-0.67	4.64E-12
Hapln4	NM_001108398	chr16:19816714-19824514	-0.67	2.56E-02
-	NA	chr16:9815114-9831562	-0.67	9.14E-03
Lgi2	NM_001107219	chr14:63184649-63216440	-0.68	3.89E-05
Rph3a	NM_133518	chr12:36678210-36754689	-0.68	0.00E+00
Ppp2r2c	NM_057116	chr14:79415486-79515898	-0.68	3.70E-07
-	NA	chr7:63207771-63553368	-0.68	2.21E-02
Grm4	NM_022666	chr20:5627738-5727071	-0.69	3.99E-03
-	NA	chr10:1717053-1739167	-0.69	1.53E-02
RGD1310110	NM_001108374	chr15:25671717-25781147	-0.69	4.49E-10
Olfm1	NM_053573	chr3:7157631-7195344	-0.70	7.47E-13
-	NA	chr1:41949103-41951153	-0.70	3.85E-02
Lrfn5	NM_001108024_2	chr6:82482088-82657877	-0.70	9.77E-03
Lppr1	NM_201271	chr5:65982027-66116330	-0.71	1.86E-02
Fbxo2	NM_053511	chr5:165229493-165244488	-0.71	1.24E-09
Chrna6	NM_057184	chr16:69011658-69019972	-0.72	0.00E+00
-	NA	chr12:16147712-16149141	-0.73	6.42E-03
Vsnl1	NM_012686	chr6:34770045-34895386	-0.73	0.00E+00
Clec2l	NM_001044233	chr4:66232232-66248873	-0.73	3.82E-08
-	NA	chr8:115218302-115218437	-0.73	2.71E-02
Cpne4	NM_001109003	chr8:109647741-110112923	-0.75	2.46E-05
Ina	NM_019128	chr1:252195871-252341026	-0.75	0.00E+00
Kcnip2	NM_020095	chr1:251008674-251032456	-0.75	9.18E-04
Rab9b	NM_001109018	chrX:124525737-124528666	-0.75	7.00E-05
Chrna3	NM_052805	chr8:58566332-58583835	-0.76	7.85E-07

Drd5	NM_012768	chr14:77766667-77769867	-0.77	1.87E-03
Nat8l	NM_001191681	chr14:82447502-82457521	-0.77	0.00E+00
Tm2d1	NM_001108670	chr5:118765692-119132248	-0.78	4.35E-06
Myt1l	NM_053888	chr6:47428706-47828681	-0.79	2.20E-07
Pou4f3	NM_001108889	chr18:35600204-35603057	-0.79	9.15E-03
Elavl2	NM_173309	chr5:111178873-111327146	-0.79	2.43E-03
-	NA	chr16:18734523-18740208	-0.79	8.09E-06
Panx2	NM_199409	chr7:127364189-127374394	-0.80	5.73E-11
Grin2a	NM_012573	chr10:5586576-6013865	-0.80	1.59E-07
-	NA	chr15:36565193-36566771	-0.80	3.99E-02
Lgi1	NM_145769	chr1:242592881-242636533	-0.80	4.28E-04
Mmp17	NM_001105925	chr12:28142755-28168449	-0.81	8.37E-03
-	NA	chr3:148643479-148652012	-0.81	3.89E-05
-	NA	chr2:110357985-110358091	-0.81	4.40E-03
Prph	NM_012633	chr7:137835964-137839928	-0.81	3.08E-10
Rprm	NM_001044276	chr3:35590170-35591873	-0.81	3.67E-02
-	NA	chr1:118717423-118720044	-0.83	3.80E-02
Mybph	NM_031813	chr13:47156108-47193837	-0.83	5.55E-06
Htr5a	NM_013148	chr4:2705390-2717506	-0.84	2.00E-03
Frem3	NM_001191699	chr19:29159197-29218214	-0.84	3.03E-02
Gabrg1	NM_080586	chr14:39876945-39953402	-0.85	8.65E-03
Camk2a	NM_012920	chr18:56878839-56951588	-0.86	3.20E-07
-	NA	chrX:110556968-110561880	-0.87	2.02E-03
Bcam	NM_031752	chr1:79067352-79081805	-0.88	1.55E-04
Slc17a6	NM_053427	chr1:101425513-101466042	-0.89	0.00E+00
Trim36	NM_001106147	chr18:40328285-40356116	-0.89	1.29E-02
-	NA	chr10:9789629-9790574	-0.89	2.23E-02
Nefm	NM_017029	chr15:47698318-47709859	-0.90	0.00E+00
Cntn2	NM_012884	chr13:45395331-45428920	-0.94	3.61E-07
Kif26b	NM_001109079	chr13:94339259-94612911	-0.95	2.48E-02
Mt3	NM_053968	chr19:11284577-11286460	-0.95	2.44E-07
Grem2	NM_001105974	chr13:90528929-90622420	-0.95	1.06E-08
Mgst3	NM_001191594	chr13:83038103-83058877	-0.96	5.55E-09
Lynx1	NM_001130546	chr7:112862546-112867792	-0.97	0.00E+00
-	NA	chrX:109833903-110331815	-0.98	4.98E-05
Sgpp2	NM_001191811	chr9:77679178-77803351	-0.98	2.01E-09
MGC114464	NM_001024909	chr7:136886369-136888833	-0.99	1.09E-05
Fstl1	NM_024369	chr11:64680815-64735716	-0.99	0.00E+00
Satb2	NM_001109306	chr9:55645140-55825653	-1.00	4.04E-02
Susd2	NM_001106381	chr20:13435227-13442685	-1.00	5.70E-03
Shh	NM_017221	chr4:2200005-2210618	-1.02	1.81E-05
Nell2	NM_031070	chr7:133758272-134080307	-1.02	0.00E+00
Calb2	NM_053988	chr19:40023057-40051991	-1.02	0.00E+00

Nell1	NM_031069	chr1:99805395-100758156	-1.03	3.78E-06
-	NA	chr2:252504694-252608499	-1.07	1.78E-09
-	NA	chr1:199736919-199747891	-1.07	4.40E-03
Gpr139	NM_001024241	chr1:177370228-177415330	-1.09	3.67E-02
Chrnb3	NM_133597	chr16:69023427-69071832	-1.10	0.00E+00
Kcnk9	NM_053405	chr7:110223532-110269195	-1.14	0.00E+00
Irx3	NM_001107413	chr19:16344771-16347445	-1.15	3.46E-02
-	NA	chr5:140059136-140085868	-1.16	0.00E+00
RGD1559613	NM_001109138	chr1:65867202-65887695	-1.18	1.46E-08
Nefl	NM_031783	chr15:47634989-47641495	-1.27	0.00E+00
Hapln1	NM_019189	chr2:19575901-19639454	-1.28	3.03E-02
Ndst4	NM_001191849	chr2:222817104-222819694	-1.28	8.09E-03
Gpr165	NM_001106582	chrX:84285376-84293665	-1.30	3.86E-08
Sec14l3	NM_022608	chr14:84699286-84710130	-1.33	1.59E-03
P2rx6	NM_012721	chr11:85426444-85441202	-1.34	2.99E-02
Aifm3	NM_001013977	chr11:85496161-85512809	-1.35	1.81E-02
Sncg	NM_031688	chr16:10025975-10030826	-1.37	0.00E+00
Syt2	NM_012665	chr13:47591094-47704070	-1.38	0.00E+00
Tfap2d	NM_001106895	chr9:17936758-18009237	-1.40	1.30E-03
Thy1	NM_012673	chr8:47027649-47032818	-1.41	0.00E+00
-	NA	chr7:110222641-110223466	-1.42	4.46E-02
Hebp2	NM_001107515	chr1:13629673-13635948	-1.42	1.04E-04
Tusc5	NM_001039163	chr10:67473528-67502815	-1.45	0.00E+00
Mogat2	NM_001109436	chr1:156516568-156540905	-1.46	4.93E-02
-	NA	chr6:44931170-44952387	-1.49	1.32E-05
-	NA	chr16:62009658-62164605	-1.49	2.60E-08
Ywhah	NM_013052	chr14:83444677-83448067	-1.52	1.86E-02
Mta1	NM_022588	chr6:137779177-137802848	-1.52	3.00E-08
Kcns3	NM_031778	chr6:34534561-34593476	-1.54	1.38E-02
Lgi3	NM_001107277	chr15:50932284-50939310	-1.57	0.00E+00
Mta1	NM_022588	chr6:137756120-137778651	-1.58	0.00E+00
Dpp10	NM_001012205	chr13:35532550-35722537	-1.58	0.00E+00
Kcnd2	NM_031730	chr4:47540608-47543454	-1.61	1.09E-09
-	NA	chr19:25807375-25811426	-1.64	9.95E-03
-	NA	chr15:88538155-88539519	-1.65	7.56E-06
Htr1d	NM_012852	chr5:155169404-155191136	-1.67	4.36E-04
Ky	NM_001108180	chr8:107472110-107510418	-1.70	1.32E-02
Cyp2s1	NM_001107495	chr1:81008061-81023746	-1.79	2.29E-06
-	NA	chr15:88533023-88537760	-1.86	0.00E+00
Kcns2	NM_023966	chr7:70277518-70283818	-1.90	0.00E+00
Scn4b	NM_001008880	chr8:48090882-48106709	-1.93	0.00E+00
Pou4f2	NM_134355	chr19:31388881-31392549	-1.94	0.00E+00
Nefh	NM_012607	chr14:85594788-85609433	-2.06	0.00E+00

-	NA	chr17:37679065-37688020	-2.06	1.79E-02
Rbpms2	NM_001173426	chr8:69907640-69919580	-2.21	0.00E+00
Irx2	NM_001039505	chr17:743563-748542	-2.22	3.17E-05
Tppp3	NM_001009639	chr19:35284037-35314432	-2.38	0.00E+00
Htr1b	NM_022225	chr8:86665505-86702552	-2.42	0.00E+00
Rasgrp2	NM_001082977	chr1:209183023-209199161	-2.47	1.92E-05
Isl2	NM_020471	chr8:59105622-59111897	-2.54	8.47E-10
Kcng1	NM_001106545	chr3:159426269-159446526	-2.58	7.51E-07
Irx4	NM_001107330	chr17:106087-116010	-2.73	0.00E+00
Kcnip4	NM_181365	chr14:66526603-66527041	-3.67	2.09E-02
-	NA	chr3:7268470-7298938	-3.98	1.84E-13

Minus symbol: unannotated; N/A: Not applicable.

Table S4. List of overlapping upregulated genes in the rat ONT and mouse ONC models

Symbol	Log_2 FC	q-value
Ecel1	5.86	1.60E-10
Tnfrsf12a	3.68	0.00E+00
Gpnmb	3.32	0.00E+00
Lgals3	2.93	9.05E-08
Hmox1	2.65	5.30E-13
Csrnp1	2.63	8.75E-08
Sox11	2.36	0.00E+00
Chac1	2.34	0.00E+00
Atf3	2.15	0.00E+00
Egr1	1.94	0.00E+00
Arid5a	1.56	1.87E-11
Stk32a	1.40	0.00E+00
Slc7a1	1.33	0.00E+00
Stmn4	1.29	0.00E+00
Asns	1.25	0.00E+00
Srxn1	1.21	0.00E+00
Vgf	1.07	0.00E+00
Cyp4v3	1.02	3.41E-03
Fam129a	0.97	5.80E-04
Mthfd2	0.95	1.34E-12
Tac1	0.94	7.76E-10
Rab15	0.92	0.00E+00
Atf5	0.90	0.00E+00
Slc7a3	0.90	2.99E-10
Clic1	0.82	3.98E-02
Rhoq	0.80	9.93E-06
Cdkn1a	0.78	1.26E-02
Phgdh	0.78	1.61E-07
Slc1a4	0.72	8.40E-11
Jun	0.70	0.00E+00
Cars	0.67	2.47E-10
Dusp1	0.66	4.86E-08
Chi3l1	0.65	4.02E-08
Tes	0.65	7.94E-04
Plekho1	0.62	2.23E-03

Table S5. Top 20 downregulated genes after ONT

Symbol	RefSeq	Gene locus	$\log_2$FC	q-value	Mean FPKM of ONT	Mean FPKM of sham
Kcnip4†	NM_181365	chr14:66526603-66527041	-3.67	2.09E-02	0.27	3.44
Irx4	NM_001107330	chr17:106087-116010	-2.73	0.00E+00	0.53	3.53
Kcng1	NM_001106545	chr3:159426269-159446526	-2.58	7.51E-07	0.12	0.71
Isl2†	NM_020471	chr8:59105622-59111897	-2.54	8.47E-10	0.33	1.92
Rasgrp2†	NM_001082977	chr1:209183023-209199161	-2.47	1.92E-05	0.55	3.03
Htr1b†	NM_022225	chr8:86665505-86702552	-2.42	0.00E+00	0.94	5.05
Tppp3†	NM_001009639	chr19:35284037-35314432	-2.38	0.00E+00	6.47	33.62
Irx2	NM_001039505	chr17:743563-748542	-2.22	3.17E-05	0.24	1.12
Rbpms2†	NM_001173426	chr8:69907640-69919580	-2.21	0.00E+00	3.12	14.40
Nefh	NM_012607	chr14:85594788-85609433	-2.06	0.00E+00	3.89	16.20
Pou4f2†	NM_134355	chr19:31388881-31392549	-1.94	0.00E+00	0.78	2.98
Scn4b	NM_001008880	chr8:48090882-48106709	-1.93	0.00E+00	0.64	2.44
Kcns2	NM_023966	chr7:70277518-70283818	-1.90	0.00E+00	0.44	1.65
Cyp2s1†	NM_001107495	chr1:81008061-81023746	-1.79	2.29E-06	0.32	1.10
Ky	NM_001108180	chr8:107472110-107510418	-1.70	1.32E-02	0.13	0.43
Htr1d†	NM_012852	chr5:155169404-155191136	-1.67	4.36E-04	0.42	1.32
Kcnd2†	NM_031730	chr4:47540608-47543454	-1.61	1.09E-09	1.97	5.50
Dpp10	NM_001012205	chr13:35532550-35722537	-1.58	0.00E+00	2.51	7.22
Mta1	NM_022588	chr6:137756120-137778651	-1.58	0.00E+00	0.61	1.82
Lgi3	NM_001107277	chr15:50932284-50939310	-1.57	0.00E+00	2.79	8.29

Red: Overlapping downregulated genes in the rat ONT and mouse ONC models. †Specifically expressed in RGCs[25].

Table S6. List of differentially expressed transcripts after ONT

Symbol	RefSeq	Gene locus	log_2FC	q-value
Ecel1	NM_021776	chr9:85939912-85947141	5.77	0.00E+00
Casp4	NM_053736	chr8:2038574-2076531	4.58	2.32E-05
-	N/A	chr16:26443686-26444348	4.23	2.64E-11
Stc2	NM_022230	chr10:16514554-16526844	4.07	1.19E-13
RGD1304592	NM_001134581	chr4:180903354-180990103	3.87	3.60E-02
Ifi27l2b	NM_206846	chr6:127733546-127735079	3.76	0.00E+00
Stbd1	NM_001013988	chr14:17031959-17035146	3.58	0.00E+00
Tnfrsf12a	NM_181086	chr10:12940402-12942373	3.57	0.00E+00
Gpnmb	NM_133298	chr4:77161283-77182621	3.39	0.00E+00
Dvl1	NM_031820	chr5:172705596-172716464	3.32	2.59E-02
Sipa1l1	NM_139330	chr6:106005229-106283658	3.10	1.71E-03
Arhgap39	NM_173122	chr7:114760183-114866892	3.01	3.71E-02
Lgals3	NM_031832	chr15:23326768-23339109	2.93	1.55E-07
Zfp362	NM_001191612	chr5:147999454-148033760	2.91	3.97E-02
-	N/A	chr11:81115015-81216154	2.90	0.00E+00
Hspb1	NM_031970	chr12:21911202-21912866	2.74	7.75E-13
Hmox1	NM_012580	chr19:13963009-13969949	2.65	9.80E-13
Acaa1a	NM_012489	chr8:124304762-124316797	2.61	2.03E-02
Lphn2	NM_134408	chr2:247060278-247722292	2.54	2.42E-02
Lcn2	NM_130741	chr3:11511400-11515051	2.52	1.27E-11
-	N/A	chr8:101018891-101129493	2.45	1.40E-05
RGD1564664	NM_001110055	chr3:78365767-78372059	2.43	0.00E+00
Csrp3	NM_057144	chr1:98601687-98620255	2.42	2.15E-08
Creb5	NM_001134621	chr4:81616078-81617489	2.39	4.90E-02
Sox11	NM_053349	chr6:45135451-45143452	2.36	0.00E+00
Chac1	NM_001173437	chr3:105864667-105873323	2.34	0.00E+00
Lrrc28	NM_001108486	chr1:122266746-122385117	2.25	1.68E-02
-	N/A	chr3:40019207-40022593	2.16	3.08E-03
Mum1	NM_001108736	chr7:10976489-10993339	2.07	6.42E-03
Serinc2	NM_001031656	chr5:149279202-149301822	1.93	6.24E-03
Eif4ebp1	NM_053857	chr16:69110555-69123897	1.90	1.98E-04
Cd24	NM_012752	chr20:47492883-47507659	1.86	1.92E-06
Dnmt3a	NM_001003958	chr6:26767283-26884349	1.84	3.06E-02
Il28ra	NM_001191868	chr5:154461480-154483067	1.79	3.40E-03
-	N/A	chr16:60118901-60136536	1.75	8.18E-03
Zhx3	NM_001047097	chr3:151522978-151671627	1.74	3.76E-04
Mical3	NM_001191085	chr4:157335111-157566479	1.66	2.00E-12
Mir431	NR_031953	chr6:134181230-134186527	1.64	3.18E-05
Zfp608	NM_001107378	chr18:50303776-50420069	1.62	7.65E-03
Tac1	NM_012666	chr4:32648884-32657616	1.60	2.19E-03
Arhgef2	NM_001012079	chr2:180742964-180777323	1.55	1.67E-07

Table S5. List of differentially expressed transcripts after ONT

Symbol	RefSeq	Gene locus	$log_2 FC$	q-value
Ecel1	NM_021776	chr9:85939912-85947141	5.77	0.00E+00
Casp4	NM_053736	chr8:2038574-2076531	4.58	2.32E-05
-	N/A	chr16:26443686-26444348	4.23	2.64E-11
Stc2	NM_022230	chr10:16514554-16526844	4.07	1.19E-13
RGD1304592	NM_001134581	chr4:180903354-180990103	3.87	3.60E-02
Ifi27l2b	NM_206846	chr6:127733546-127735079	3.76	0.00E+00
Stbd1	NM_001013988	chr14:17031959-17035146	3.58	0.00E+00
Tnfrsf12a	NM_181086	chr10:12940402-12942373	3.57	0.00E+00
Gpnmb	NM_133298	chr4:77161283-77182621	3.39	0.00E+00
Dvl1	NM_031820	chr5:172705596-172716464	3.32	2.59E-02
Sipa1l1	NM_139330	chr6:106005229-106283658	3.10	1.71E-03
Arhgap39	NM_173122	chr7:114760183-114866892	3.01	3.71E-02
Lgals3	NM_031832	chr15:23326768-23339109	2.93	1.55E-07
Zfp362	NM_001191612	chr5:147999454-148033760	2.91	3.97E-02
-	N/A	chr11:81115015-81216154	2.90	0.00E+00
Hspb1	NM_031970	chr12:21911202-21912866	2.74	7.75E-13
Hmox1	NM_012580	chr19:13963009-13969949	2.65	9.80E-13
Acaa1a	NM_012489	chr8:124304762-124316797	2.61	2.03E-02
Lphn2	NM_134408	chr2:247060278-247722292	2.54	2.42E-02
Lcn2	NM_130741	chr3:11511400-11515051	2.52	1.27E-11
-	N/A	chr8:101018891-101129493	2.45	1.40E-05
RGD1564664	NM_001110055	chr3:78365767-78372059	2.43	0.00E+00
Csrp3	NM_057144	chr1:98601687-98620255	2.42	2.15E-08
Creb5	NM_001134621	chr4:81616078-81617489	2.39	4.90E-02
Sox11	NM_053349	chr6:45135451-45143452	2.36	0.00E+00
Chac1	NM_001173437	chr3:105864667-105873323	2.34	0.00E+00
Lrrc28	NM_001108486	chr1:122266746-122385117	2.25	1.68E-02
-	N/A	chr3:40019207-40022593	2.16	3.08E-03
Mum1	NM_001108736	chr7:10976489-10993339	2.07	6.42E-03
Serinc2	NM_001031656	chr5:149279202-149301822	1.93	6.24E-03
Eif4ebp1	NM_053857	chr16:69110555-69123897	1.90	1.98E-04
Cd24	NM_012752	chr20:47492883-47507659	1.86	1.92E-06
Dnmt3a	NM_001003958	chr6:26767283-26884349	1.84	3.06E-02
Il28ra	NM_001191868	chr5:154461480-154483067	1.79	3.40E-03
-	N/A	chr16:60118901-60136536	1.75	8.18E-03
Zhx3	NM_001047097	chr3:151522978-151671627	1.74	3.76E-04
Mical3	NM_001191085	chr4:157335111-157566479	1.66	2.00E-12
Mir431	NR_031953	chr6:134181230-134186527	1.64	3.18E-05
Zfp608	NM_001107378	chr18:50303776-50420069	1.62	7.65E-03
Tac1	NM_012666	chr4:32648884-32657616	1.60	2.19E-03
Arhgef2	NM_001012079	chr2:180742964-180777323	1.55	1.67E-07

Pim1	NM_017034	chr20:7817008-7822801	1.52	1.01E-08
Stmn4	NM_019176	chr15:45779151-45797534	1.44	5.01E-03
C1qtnf1	NM_001007675	chr10:108428236-108453209	1.43	2.59E-03
-	N/A	chr1:1588230-1594890	1.41	2.19E-03
Sema4c	NM_001106902	chr9:35492949-35502474	1.41	3.78E-02
RGD1566319	NM_001109358	chr5:152639880-152655830	1.40	1.46E-02
Stmn4	NM_019176	chr15:45779151-45797534	1.37	0.00E+00
Ramp3	NM_020100	chr14:87417276-87434785	1.32	3.07E-04
Ash2l	NM_001106089	chr16:70617704-70663900	1.26	2.87E-04
Nlgn1	NM_053868	chr2:111187910-111409259	1.24	4.61E-02
-	N/A	chr9:85988788-85993978	1.24	0.00E+00
Clic1	NM_001002807	chr20:3837345-3842970	1.24	1.00E-10
Stmn4	NM_019176	chr15:45779151-45797534	1.23	2.91E-02
Rfx3	NM_001012172	chr1:231336479-231601819	1.22	4.40E-02
PVR	NM_017076	chr1:79212729-79229069	1.21	3.88E-04
Srxn1	NM_001047858	chr3:142457296-142463397	1.21	0.00E+00
Slc26a2	NM_057127	chr18:57165916-57184838	1.20	2.40E-03
-	N/A	chr16:31661383-31957766	1.12	1.06E-02
-	N/A	chr7:57333751-57337699	1.11	6.43E-04
Rnf148	NM_001191082	chr4:49658565-50206224	1.10	1.81E-03
Cth	NM_017074	chr2:256056567-256082450	1.10	4.22E-03
-	N/A	chr20:3961364-3964119	1.09	4.33E-09
Chka	NM_017127	chr1:206368768-206418506	1.07	4.17E-02
Lmna	NM_001002016	chr2:180595679-180616699	1.07	1.83E-02
Shmt2	NM_001008322	chr7:67486345-67499565	1.07	5.66E-04
Hspb8	NM_053612	chr12:41407000-41421553	1.07	2.48E-10
-	N/A	chr5:157619679-157649295	1.05	4.80E-02
Fam13b	NM_001106158	chr18:26911307-26983446	1.05	2.48E-02
RGD1359634	NM_001007708	chr7:117087031-117122728	1.05	5.51E-04
Plag1	NM_001008316	chr5:17211631-17261459	1.04	1.42E-02
Stim2	NM_001105750	chr14:61631936-61759973	1.03	8.24E-04
C2cd4c	NM_001109285	chr7:11608875-11612166	1.01	9.18E-03
Nos1	NM_052799	chr12:39811703-39893785	1.00	3.60E-07
Rhoq	NM_053522	chr6:10414284-10474868	1.00	1.19E-13
-	N/A	chr7:26147257-26154852	1.00	3.86E-02
Utp15	NM_001107647	chr2:28834447-28864817	1.00	4.68E-02
Kcnk12	NM_022292	chr6:11277526-11355065	0.98	2.52E-08
Tac1	NM_012666	chr4:32648884-32657616	0.97	3.55E-03
Lonrf3	NM_001191585	chrX:8562796-8566005	0.97	7.72E-03
Fam129a	NM_022242	chr13:66467068-66621851	0.97	8.67E-04
Gadd45g	NM_001077640	chr17:19230900-19232842	0.97	2.34E-13
Bspry	NM_022261	chr5:79471064-79498789	0.96	6.43E-04
Rsrc1	NM_001014172_2	chr2:156893802-157215064	0.96	2.18E-05

Mafk	NM_145673	chr12:15312413-15323194	0.95	2.68E-04
Sppl2b	NM_001014200	chr7:10354657-10369703	0.95	9.59E-03
Wls	NM_199408	chr2:258014378-258128850	0.94	1.59E-06
Sars	NM_001007606	chr2:203995598-204011329	0.93	5.07E-03
Rab15	NM_198749	chr6:99378936-99403488	0.92	0.00E+00
Atf5	NM_172336	chr1:95284083-95303547	0.90	0.00E+00
Slc7a3	NM_017217	chrX:89159609-89165672	0.90	5.56E-10
Slc6a8	NM_017348	chrX:159570631-159580110	0.90	0.00E+00
-	N/A	chr1:208476329-208480254	0.87	1.74E-02
-	N/A	chr2:127556375-127642220	0.87	4.31E-02
Nipa1	NM_001107519	chr1:107368206-107433994	0.84	3.63E-02
Anxa2	NM_019905	chr8:73897548-73934051	0.84	3.90E-11
Clic1	NM_001002807	chr20:3498999-3499328	0.82	4.95E-02
Suv39h2	NM_001108883	chr17:85873810-85896680	0.79	1.26E-02
Klf6	NM_031642	chr17:75576214-75585151	0.79	9.84E-10
Phgdh	NM_031620	chr2:193147938-193177099	0.78	2.79E-07
Cdkn1a	NM_080782	chr20:7385786-7386778	0.78	1.70E-02
Tac1	NM_012666	chr4:32648884-32657616	0.77	1.15E-03
-	N/A	chr4:163620377-163642092	0.74	1.49E-03
-	N/A	chr1:208476329-208480254	0.74	1.18E-12
Abcb1a	NM_133401	chr4:21497686-21796596	0.72	1.37E-02
Setd7	NM_001109558	chr2:140408843-140457440	0.71	0.00E+00
Htr2a	NM_017254	chr15:55462700-55535175	0.69	3.58E-03
Flrt3	NM_001126291	chr3:128921577-128935096	0.68	6.44E-07
-	N/A	chr10:35904933-35910391	0.68	1.02E-02
Tnik	NM_001106422	chr2:115597332-116009522	0.67	3.92E-03
Tnik	NM_001106422	chr2:115597332-116009522	0.67	6.39E-07
Dusp1	NM_053769	chr10:16942654-16945693	0.66	8.55E-08
Luc7l2	NM_001107853	chr4:66121260-66182194	0.66	1.70E-02
Tes	NM_001039344	chr4:42680546-42719249	0.65	1.19E-03
Eprs	NM_001024238	chr13:101374690-101448389	0.65	3.65E-08
Pde10a	NM_022236	chr1:46388571-46855563	0.65	9.34E-03
Gpr137c	NM_001134541	chr15:21029771-21167044	0.64	6.77E-04
-	N/A	chr4:57071285-57087830	0.64	2.92E-02
Sema3c	NM_001106578	chr4:13739550-13916902	0.63	2.76E-02
Ddit3	NM_024134	chr7:67223738-67269924	0.62	5.91E-03
Tnik	NM_001106422	chr2:115597332-116009522	0.61	6.06E-03
RGD1306119	NM_001169116	chr6:108200993-108270671	0.61	3.85E-05
Eef1a2	NM_012660	chr3:170295678-170307673	0.60	0.00E+00
Crot	NM_031987	chr4:22023632-22073718	0.59	1.50E-02
Zfp192	NM_001100574	chr17:50680572-50708728	0.59	3.85E-02
Kcnip4	NM_181365	chr14:67202459-67453875	-0.59	1.15E-06
Sorl1	NM_053519	chr8:44958443-45101939	-0.59	0.00E+00

Cdh13	NM_138889	chr19:48506880-49575355	-0.59	1.94E-03
Necab2	NM_133415	chr19:49691464-49718720	-0.60	2.52E-03
Kcnc1	NM_012856	chr1:96928121-96970357	-0.60	4.02E-02
Matk	NM_021859	chr7:9936608-9946664	-0.61	1.43E-03
Olfm1	NM_053573	chr3:7157631-7195344	-0.62	0.00E+00
Lgi1	NM_145769	chr1:242592881-242636533	-0.62	2.14E-02
Atl1	NM_001009831	chr6:91884703-92077631	-0.62	4.47E-06
-	N/A	chr5:61982036-62090680	-0.63	6.31E-04
Ncdn	NM_053543	chr5:146157061-146260551	-0.63	1.19E-08
Snca	NM_019169	chr4:89613822-89723335	-0.63	0.00E+00
Pcdh7	NM_001004087	chr14:57257427-57326354	-0.63	8.26E-06
Bend6	NM_001108792	chr9:32607617-32656101	-0.63	1.05E-04
Magee1	NM_001079891	chrX:93191779-93195782	-0.64	2.10E-11
Gnal	NM_001191836	chr18:63595252-63756786	-0.65	2.38E-04
-	N/A	chr2:181166191-181168360	-0.65	4.57E-03
Ndrg4	NM_031967	chr19:9808014-9843959	-0.65	1.46E-09
Irx6	NM_001102413	chr19:15432294-15440464	-0.65	1.41E-02
Slc35f1	NM_001109338	chr20:31209336-31770161	-0.65	2.93E-10
Kcnab2	NM_017304	chr5:169570781-169797466	-0.65	4.62E-02
Ampd2	NM_001101681	chr2:203633205-203645819	-0.65	3.94E-04
Nptx1	NM_153735	chr10:108918366-108927773	-0.66	1.86E-02
Mmp15	NM_001106168	chr19:10122504-10143923	-0.66	2.40E-03
Spock3	NM_001107310	chr16:29751354-29751958	-0.67	2.75E-02
Nptx1	NM_153735	chr10:108918366-108927773	-0.67	6.76E-08
-	N/A	chr7:63207771-63553368	-0.68	2.92E-02
Rph3a	NM_133518	chr12:36678210-36754689	-0.68	4.44E-08
Grm4	NM_022666	chr20:5627738-5727071	-0.69	5.67E-03
-	N/A	chr10:1717053-1739167	-0.69	2.04E-02
RGD1310110	NM_001108374	chr15:25671717-25781147	-0.69	8.41E-10
Adora1	NM_017155	chr13:47156108-47193837	-0.70	2.56E-08
-	N/A	chr1:41949103-41951153	-0.70	4.80E-02
Lrfn5	NM_001108024_2	chr6:82482088-82657877	-0.70	1.34E-02
Lppr1	NM_201271	chr5:65982027-66116330	-0.71	2.48E-02
Fbxo2	NM_053511	chr5:165229493-165244488	-0.71	2.31E-09
Chrna6	NM_057184	chr16:69011658-69019972	-0.72	0.00E+00
-	N/A	chr12:16147712-16149141	-0.73	8.89E-03
Vsnl1	NM_012686	chr6:34770045-34895386	-0.73	0.00E+00
Clec2l	NM_001044233	chr4:66232232-66248873	-0.73	6.76E-08
-	N/A	chr8:115218302-115218437	-0.73	3.56E-02
Ina	NM_019128	chr1:252195871-252341026	-0.75	0.00E+00
Adam23	NM_001029899	chr9:62105884-62253219	-0.75	2.93E-04
Cnnm1	NM_001107593	chr1:247349893-247408840	-0.75	7.65E-03
Rab9b	NM_001109018	chrX:124525737-124528666	-0.75	1.13E-04

Chrna3	NM_052805	chr8:58566332-58583835	-0.76	1.31E-06
Drd5	NM_012768	chr14:77766667-77769867	-0.77	2.74E-03
Nat8l	NM_001191681	chr14:82447502-82457521	-0.77	0.00E+00
Pou4f3	NM_001108889	chr18:35600204-35603057	-0.79	1.26E-02
-	N/A	chr9:52092314-52217783	-0.79	3.71E-02
-	N/A	chr15:36565193-36566771	-0.80	4.96E-02
-	N/A	chr3:148643479-148652012	-0.81	6.43E-05
-	N/A	chr2:110357985-110358091	-0.81	6.21E-03
Rprm	NM_001044276	chr3:35590170-35591873	-0.81	4.61E-02
Slc4a3	NM_017049	chr9:74823032-74840975	-0.82	3.78E-02
-	N/A	chr1:118717423-118720044	-0.83	4.74E-02
Grin1	NM_017010	chr3:3453748-3480741	-0.83	1.92E-02
Htr5a	NM_013148	chr4:2705390-2717506	-0.84	2.93E-03
Frem3	NM_001191699	chr19:29159197-29218214	-0.84	3.90E-02
Trim32	NM_001012103	chr5:82124669-83128667	-0.85	2.18E-02
Palm	NM_130829	chr7:11399536-11426111	-0.86	4.64E-04
RGD1559613	NM_001109138	chr1:65867202-65887695	-0.86	1.72E-04
Mmp17	NM_001105925	chr12:28142755-28168449	-0.87	2.59E-03
Lynx1	NM_001130546	chr7:112862546-112867792	-0.87	0.00E+00
-	N/A	chrX:110556968-110561880	-0.87	2.95E-03
Lrrn1	NM_001037363	chr4:142969378-143009452	-0.89	7.43E-05
Usp15	NM_145184	chr7:62867022-62961472	-0.89	1.32E-02
Slc17a6	NM_053427	chr1:101425513-101466042	-0.89	0.00E+00
Trim36	NM_001106147	chr18:40328285-40356116	-0.89	1.74E-02
Nlgn3	NM_134336	chrX:89374995-89404172	-0.89	2.08E-03
-	N/A	chr10:9789629-9790574	-0.89	2.94E-02
Nefm	NM_017029	chr15:47698318-47709859	-0.90	0.00E+00
Vamp1	NM_013090	chr4:161333442-161354071	-0.92	1.90E-06
Vwa5b2	NM_001134535	chr11:82529324-82552952	-0.92	1.41E-02
Nell2	NM_031070	chr7:133758272-134080307	-0.92	0.00E+00
Prph	NM_012633	chr7:137835964-137839928	-0.92	1.65E-03
Camk2a	NM_012920	chr18:56878839-56951588	-0.94	5.95E-11
Camk2a	NM_012920	chr18:56878839-56951588	-0.95	2.94E-03
Mt3	NM_053968	chr19:11284577-11286460	-0.95	4.27E-07
Grem2	NM_001105974	chr13:90528929-90622420	-0.95	1.89E-08
Cpne4	NM_001109003	chr8:109647741-110112923	-0.96	3.51E-05
Mgst3	NM_001191594	chr13:83038103-83058877	-0.96	1.01E-08
-	N/A	chrX:109833903-110331815	-0.98	8.19E-05
Sgpp2	NM_001191811	chr9:77679178-77803351	-0.98	3.65E-09
Calb2	NM_053988	chr19:40023057-40051991	-0.99	0.00E+00
MGC114464	NM_001024909	chr7:136886369-136888833	-0.99	1.81E-05
Fstl1	NM_024369	chr11:64680815-64735716	-0.99	0.00E+00
Srsf7	NM_001039035	chr6:3097295-3104609	-1.00	4.96E-02

Olfm1	NM_053573	chr3:7157631-7195344	-1.00	4.22E-02
Shh	NM_017221	chr4:2200005-2210618	-1.02	2.98E-05
Esf1	NM_001100771	chr3:128283412-128353763	-1.03	1.18E-02
Panx2	NM_199409	chr7:127364189-127374394	-1.04	0.00E+00
Mir350	NR_031794	chr13:92502352-92612085	-1.07	2.11E-04
-	N/A	chr2:252504694-252608499	-1.07	3.21E-09
-	N/A	chr1:199736919-199747891	-1.07	6.21E-03
Calb2	NM_053988	chr19:40023057-40051991	-1.08	0.00E+00
Gpr139	NM_001024241	chr1:177370228-177415330	-1.09	4.61E-02
Chst11	NM_001108079	chr7:22718327-22950259	-1.10	4.58E-02
Susd2	NM_001106381	chr20:13435227-13442685	-1.10	2.95E-03
Cd99l2	NM_134459	chr15:5673483-5719187	-1.11	1.74E-05
Tppp	NM_001108461	chr1:30063540-30087600	-1.11	4.40E-02
Pc	NM_012744	chr1:207112823-207212853	-1.11	4.02E-02
Lnpep	NM_133574	chr1:56055322-56151458	-1.12	4.39E-02
Kcnk9	NM_053405	chr7:110223532-110269195	-1.14	0.00E+00
Lin54	NM_001100564	chr14:10381771-10439930	-1.14	4.59E-02
Irx3	NM_001107413	chr19:16344771-16347445	-1.15	4.39E-02
Nell1	NM_031069	chr1:99805395-100758156	-1.17	9.82E-08
Cntn2	NM_012884	chr13:45395331-45428920	-1.19	1.34E-07
Cnot6l	NM_001108355	chr14:14923797-15016325	-1.20	2.48E-02
-	N/A	chr16:62009658-62164605	-1.21	4.15E-02
Nefl	NM_031783	chr15:47634989-47641495	-1.21	2.32E-09
-	N/A	chr4:185677882-185827929	-1.27	1.83E-02
-	N/A	chr5:140059136-140085868	-1.28	0.00E+00
Hapln1	NM_019189	chr2:19575901-19639454	-1.28	3.90E-02
Ndst4	NM_001191849	chr2:222817104-222819694	-1.28	1.11E-02
Gpr165	NM_001106582	chrX:84285376-84293665	-1.30	6.76E-08
Hpse	NM_022605_2	chr14:10147048-10185421	-1.30	2.25E-03
Syt1	NM_001033680	chr7:47240666-47796369	-1.31	3.43E-06
Camk2g	NM_133605	chr15:3729101-3787934	-1.35	7.62E-04
Kcnd2	NM_031730	chr4:48029879-48048838	-1.37	6.60E-09
Wdr20a	NM_001100894	chr6:135460190-135531188	-1.38	2.27E-02
Hebp2	NM_001107515	chr1:13629673-13635948	-1.42	1.67E-04
Inpp4b	NM_053917	chr19:27717037-28480670	-1.45	2.44E-02
Grin2a	NM_012573	chr10:5586576-6013865	-1.47	2.44E-04
Qk	NM_001115021	chr1:44922561-45031145	-1.47	1.41E-02
-	N/A	chr6:44931170-44952387	-1.49	2.18E-05
-	N/A	chr1:241293119-241498166	-1.50	9.54E-03
Ywhah	NM_013052	chr14:83444677-83448067	-1.52	2.48E-02
Mta1	NM_022588	chr6:137779177-137802848	-1.52	5.27E-08
Kcns3	NM_031778	chr6:34534561-34593476	-1.54	1.86E-02
Car5b	NM_001005551	chrX:51229138-51291388	-1.54	4.23E-02

Lgi3	NM_001107277	chr15:50932284-50939310	-1.57	0.00E+00
-	N/A	chr9:77595682-77601103	-1.58	3.72E-02
Kcnd2	NM_031730	chr4:47540608-47543454	-1.61	2.03E-09
Rtkn	NM_184046	chr4:117347225-117363963	-1.63	3.23E-03
-	N/A	chr16:62009658-62164605	-1.65	3.70E-10
Dpp10	NM_001012205	chr13:35532550-35722537	-1.65	0.00E+00
Ky	NM_001108180	chr8:107472110-107510418	-1.70	1.79E-02
-	N/A	chr1:205980405-206016673	-1.71	5.08E-03
Cyp2s1	NM_001107495	chr1:81008061-81023746	-1.79	3.72E-06
-	N/A	chr15:88533023-88537760	-1.83	2.79E-09
Lrrc51	NM_001106284	chr1:159372942-159393171	-1.87	8.89E-03
Kcnip2	NM_020095	chr1:251008674-251032456	-1.88	6.93E-04
Kcns2	NM_023966	chr7:70277518-70283818	-1.90	0.00E+00
-	N/A	chr15:88533023-88537760	-1.90	0.00E+00
Scn4b	NM_001008880	chr8:48090882-48106709	-1.93	0.00E+00
Pou4f2	NM_134355	chr19:31388881-31392549	-1.94	0.00E+00
Tfap2d	NM_001106895	chr9:17936758-18009237	-1.98	1.20E-06
-	N/A	chr5:118765692-119132248	-2.11	4.71E-06
Rin2	NM_001107786	chr3:134284525-134503896	-2.22	7.72E-03
Irx2	NM_001039505	chr17:743563-748542	-2.22	5.25E-05
Isl2	NM_020471	chr8:59105622-59111897	-2.34	2.04E-08
Htr1b	NM_022225	chr8:86665505-86702552	-2.35	0.00E+00
P2rx6	NM_012721	chr11:85426444-85441202	-2.37	4.48E-02
Chn1	NM_032083	chr3:56144154-56368079	-2.43	4.31E-02
Htr1b	NM_022225	chr8:86665505-86702552	-2.44	1.18E-02
Tppp3	NM_001009639	chr19:35284037-35314432	-2.51	0.00E+00
Rasgrp2	NM_001082977	chr1:209183023-209199161	-2.53	2.03E-04
Rbpms2	NM_001173426	chr8:69907640-69919580	-2.53	0.00E+00
Kcng1	NM_001106545	chr3:159426269-159446526	-2.58	1.26E-06
Smad9	NM_138872	chr2:143981061-144034074	-2.60	1.74E-02
Exoc2	NM_134414	chr17:39951103-40125373	-2.63	1.69E-02
Irx4	NM_001107330	chr17:106087-116010	-2.73	0.00E+00
Pls3	NM_031084	chrX:29868930-29965164	-2.98	1.00E-02
-	N/A	chr8:101018891-101129493	-3.08	3.03E-04
-	N/A	chr7:129336889-129456789	-3.24	1.60E-03
-	N/A	chr12:25518246-26168766	-3.32	3.03E-02
Rnf31	NM_001108868	chr15:33722399-33746151	-3.33	2.10E-03
Kcnip4	NM_181365	chr14:66526603-66527041	-3.67	2.77E-02

Differences were considered significant when the q-value was < 0.05 and |FC| was > 1.5
Potentially novel isoforms are highlightened in green.
Minus symbol: unannotated; N/A: Not applicable.
